# Arbuscular mycorrhizal fungi improve selenium uptake by modulating root transcriptome of rice (Oryza sativa L.)

**DOI:** 10.3389/fpls.2023.1242463

**Published:** 2023-09-20

**Authors:** Yan Qin, Qiuliang Cai, Yiting Ling, Xue Chen, Jingmao Xu, Guirong Huang, Shanhe Liang, Xiu Yuan, Xiao Mu Yang, Dan Lu, Xueli Wang, Yanyan Wei

**Affiliations:** ^1^ State Key Laboratory for Conservation and Utilization of Subtropical Agri–bioresources, Guangxi Key Laboratory for Agro-Environment and Agro-Products Safety, National Demonstration Center for Experimental Plant Science Education, College of Agriculture, Guangxi University, Nanning, China; ^2^ Industrial College of Subtropical Characteristic Agriculture, Agriculture and Food Engineering College, Baise University, Baise, China; ^3^ Guangxi Eco-engineering Vocational & Technical College, Liuzhou, China; ^4^ Liuzhou Railway Vocational Technical College, Liuzhou, China

**Keywords:** selenium, funneliformis mosseae, rice, ionomics, WGCNA

## Abstract

Although selenium (Se) is an essential trace element in humans, the intake of Se from food is still generally inadequate throughout the world. Inoculation with arbuscular mycorrhizal fungi (AMF) improves the uptake of Se in rice (*Oryza sativa* L.). However, the mechanism by which AMF improves the uptake of Se in rice at the transcriptome level is unknown. Only a few studies have evaluated the effects of uptake of other elements in rice under the combined effects of Se and AMF. In this study, Se combined with the AMF *Funneliformis mosseae* (Fm) increased the biomass and Se concentration of rice plants, altered the pattern of ionomics of the rice roots and shoots, and reduced the antagonistic uptake of Se with nickel, molybdenum, phosphorus, and copper compared with the treatment of Se alone, indicating that Fm can enhance the effect of fertilizers rich in Se. Furthermore, a weighted gene co-expression network analysis (WGCNA) showed that the hub genes in modules significantly associated with the genes that contained Se and were related to protein phosphorylation, protein serine/threonine kinase activity, membrane translocation, and metal ion binding, suggesting that the uptake of Se by the rice roots may be associated with these genes when Fm and Se act in concert. This study provides a reference for the further exploration of genes related to Se uptake in rice under Fm treatment.

## 1 Introduction

Selenium (Se) is an essential trace element for human health and plays critical roles in the immune system, antioxidant defense, and redox homeostasis by acting as the catalytic center of several selenoproteins, including glutathione peroxidase and thioredoxin reductase (Gupta and Gupta, 2016). Humans require approximately 50-55 μg of dietary Se per day for health benefits according to the World Health Organization (WHO). However, the dietary supply of Se in people in some regions of China is below the suggested level (Dinh et al., 2018). An insufficient intake of Se is associated with Keshan disease, Kashin-Beck disease, heart disease, hyperthyroidism, and cancer (Shahid et al., 2018). Dietary supplementation with Se is relatively safe and efficient.

Rice (*Oryza sativa* L.) is one of the most important crops worldwide. In addition, rice enriched with Se is a safe and reliable source of this nutrient (D’Amato et al., 2020). Although Se is not an essential element for the growth of rice, its concentration in this crop is related to the concentration of Se in the planted soil and the agronomic measures used to grow rice (Yang et al., 2021a; Lyu et al., 2022). As a result, Se fertilizer not only enriches rice with Se but also increases yields. However, the overapplication of Se fertilizers is associated with some environmental risks, such as the contamination of soil and water, toxicity to plants and animals, and bioaccumulation in the food chain (Lemly, 1997; Gupta and Gupta, 2016; Hasanuzzaman et al., 2020). Therefore, changing the method of application and the form of Se fertilizer or simultaneously using other enhancers, such as an environmentally friendly microbial fertilizer, can improve the availability of this element in Se fertilizer (Chen et al., 2020; Yang et al., 2021b; Wang et al., 2022; Xian et al., 2022; Yuan et al., 2022). Plants primarily rely on their roots to absorb Se from the soil, and the roots can take up both inorganic Se (selenate [SeVI] and selenite [SeIV]) and organic Se [Se-amino acids, including selenocysteine (SeCys), selenomethionine (SeMet) and methylselenocysteine (MeSeCys)] (Lima et al, 2018). No specialized transporters have been identified for the absorption of selenium. However, sulfate transporters (*SULTRs*), aquaporin (*NIP2*), amino acid permease (*LHT1*), and the P transporter have been found to be associated with the uptake of Se (Trippe and Pilon-Smits, 2021). The uptake of Se by the roots is not only related to the physicochemical properties of the soil but also closely linked to soil microorganisms (Feng et al., 2023). Previous research has shown that microorganisms in the soil can not only change the distribution and speciation of Se in the soil through their own secretions, such as formic, citric, acetic, oxalic, lactic, malonic, and succinic acids (Dinh et al., 2017), but also influence the physiological activity of plant roots, thereby facilitating their uptake of Se (Ye et al., 2020). Thus, the simultaneous application of microorganisms in the production of Se-enriched rice can improve the utilization of this element in Se fertilizer. Since the metabolism of root ions also affects plant growth and development (Griffiths and York, 2020), the question of whether Se fertilizer and microbial application affects ion metabolism in the roots is of interest. Gui et al. (2022) showed that the interactions between a nutrient and Se, which may be synergistic or antagonistic, can influence the uptake and metabolism of ions in plants. But there have been relatively few studies on the effects of Se and microorganisms on the metabolism of ions by plant roots.

Arbuscular mycorrhizal fungi (AMF) are key elements of soil microbial communities that can form symbiotic relationships with the roots of most terrestrial plants, including rice (Bernaola et al., 2018). The symbiotic partnership between AMF and its host plants primarily consists of the transfer of carbon from host plants to AMF in the form of sugars and lipids in exchange for macroelements, such as phosphorus (P) and nitrogen (N) (Jiang et al., 2017; Luginbuehl et al., 2017; Xie et al., 2022). AMF will promote the ability of plants to take up macroelements and micronutrients from the soil (Kabir et al., 2020; Khan et al., 2022). Previous studies showed that inoculation with mycorrhizae can improve the uptake of Se in soybean (*Glycine max (Linn.)* Merr) and winter wheat (*Triticum aestivum* L. cv. Xiaoyan 22) (Bamberg et al., 2019; Wu et al., 2022; Zhang et al., 2022);. Furthermore, AMF can improve the efficiency of uptake of Se fertilizer in rice (Chen et al., 2020). However, the mechanisms used by Se fertilizer combined with AMF inoculation to affect ionomics and the uptake and translocation of Se in rice roots remain unclear.

In this study, ionomics were used to analyze the roots and shoots of rice that had been inoculated with the AMF *Funneliformis mosseae* (Fm) and fertilized with Se. The roots were used for a transcriptome analysis to identify the key genes related to ion uptake. Modules with a high correlation between Se and ionic metabolism were detected using a weighted gene co-expression network analysis (WGCNA). Therefore, this study may provide insights into the effects of Se fertilization and AMF inoculation on other nutrients in plants and provide a basis to explore methods to use to produce high-quality rice enriched with Se.

## 2 Materials and methods

### 2.1 Plant cultivation and inoculation

The seeds of rice (*Oryza sativa* L. cv. Xinliangyou 6) were sterilized with 10% hydrogen peroxide (H_2_O_2_) for 10 min and then rinsed. The seeds were placed in a plastic mesh that was floated on 0.5 mol L^-1^ calcium sulfate (CaSO_4_) in a container and then covered with aluminum foil for 2 d. The pre-germinated rice seeds were planted in pots filled with approximately 7 kg of a steam-sterilized mixture of soil-quartz and sand (1:3) (w/w). Soil was collected from the 0 to 20 cm horizon in a paddy field at the Guangxi University (Nanning, China). The soil had a pH of 5.93 and contained 20.88 g kg^-1^ organic matter, 1.92 g kg^-1^ total nitrogen, 1.67 g kg^-1^ total phosphorus, 18.56 g kg^-1^ total potassium, 54.12 mg kg^-1^ available phosphorus, 174.03 mg kg^-1^ available potassium, 236.25 mg kg^-1^ alkali hydrolyzed nitrogen, 20.13 cmol kg^-1^ cation exchange capacity, and 0.61 mg kg^-1^ total Se.

*Funneliformis mosseae* BGC NM02A (Fm) (supplied by Bank of Glomeromycota in China) spores and maize (*Zea mays* L. cv. Zhengda 999) plants were incubated in autoclaved fine sand for 2 months, and the sand was collected as inoculum (Ma et al., 2022a). The pots were divided into four treatments as follows: (1) non-mycorrhizal treatment (Ck) (50 g of sterile inoculum per kg of soil was added in pots); (2) AMF inoculation treatment (Fm) (50 g of inoculum of the Fm) per kg of soil was added to the pots); (3) Se treatment (Se) (the pots were added an additional 0.5 mg of Se [Na_2_SeO_3_] per kilogram of soil); and (4) the co-application of AMF and Se (Se+Fm) (the pots were inoculated with 50 g of Fm inoculum and were added an additional 0.5 mg of Se per kilogram of soil). Each treatment had three replicates with one pot for each replicate, and each pot contained six rice seedlings. The seedlings used in these study were 30 days old and approximately 12-14 cm high. The pots were placed in a greenhouse at the Guangxi University with the following conditions during the plant growth period: day/night temperatures of 28°C/22°C, 14 h/10 h day/night cycle, and 1,600 Lux, and distilled water was added to maintain the soil moisture.

The rice plants were harvested after 2 months when the AM fungi had fully colonized the roots (Campo et al., 2020). The plant samples were divided into their roots and aboveground parts, washed with tap water, passed through ionized water, and weighed. Some roots were stored at - 20°C to determine the rate of AMF colonization, while others were placed in a perforated centrifugal tube and quickly passed through liquid nitrogen for RNA extraction. Some shoots and roots were cut, oven-dried at 105°C for 30 min, and dried to a constant weight at 55°C. The dry shoots and roots were ground into a powder using a ball miller (MM400, RETSCH, Haan, Germany) to analyze the concentration of Se.

### 2.2 Quantitative evaluation of AMF colonization

The fresh roots were stained with trypan blue to determine the amount of AMF colonization using measurements of root morphology (Li et al., 2011). Briefly, the root samples were placed in 10% (w/v) potassium hydroxide (KOH) at 90°C for 30 min, washed three times with deionized water for 20 min, and finally soaked in fresh alkaline H_2_O_2_ (30 mL of 10% H_2_O_2_, 3 mL of ammonium hydroxide [NH_4_OH], and 567 mL of deionized water) for 10 min. The roots were rinsed with deionized water and immersed in 1% HCl at room temperature for 1 min before staining with 0.05% (w/v) trypan blue in lactoglycerol (lactic acid:glycerol:deionized water [1:1:1]) at 90°C for 20 min. The roots were washed with deionized water and stored in lactoglycerol at 4°C for at least 24 h before further analyses. The colonization of AMF on 30 root fragments (1.0 cm long) was quantified using a microscope.

### 2.3 Analysis of ion concentrations

The dry powder samples were digested with nitric acid (HNO_3_):H_2_O_2_ (v/v, 4:1) in a closed-vessel microwave oven (MARS 6240/50, CEM Corporation, Matthews, NC, USA) to determine the concentrations of magnesium (Mg), P, calcium (Ca), potassium (K), iron (Fe), manganese (Mn), molybdenum (Mo), boron (B), copper (Cu), zinc (Zn), aluminum (Al), silicon (Si), nickel (Ni), and Se using inductively coupled mass spectrometry (ICP-MS) (NEXION 350X, PerkinElmer Life Science Incorporated, Waltham, MA, USA) as previously described (Chen et al., 2020). The standard references of celery (*Apium graveolens* Linn.) sample GBW10229 and rice sample GBW 10045 were digested and analyzed as a technical control. The recoveries of ions in the standard celery and rice samples were 95.35%–101.89%.

### 2.4 RNA isolation, sequencing, and analysis

The TRIzol reagent (Invitrogen, Carlsbad, CA, USA) was used to separately extract the total RNA from root tissues. It was then purified using the Plant RNA Purification Reagent (Invitrogen). A Bioanalyzer 2100 (Agilent Technologies, Santa Clara, CA, USA) was used to determine the concentration of RNA. First, 1 µg of total RNA was used to prepare an RNA-Seq transcriptome library using a TruSeq RNA sample preparation kit from Illumina (San Diego, CA, USA). Briefly, mRNA was isolated by the polyA selection method using oligo (dT) beads and then fragmented into short fragments. Secondly, a SuperScript double-stranded cDNA synthesis kit (Invitrogen) with random hexamer primers (Illumina) was used to synthesize cDNA. The cDNA was subjected to end-repair, phosphorylation and ‘A’ base addition according to the manufacturer’s instructions. The libraries were size-selected for cDNA target fragments of 200–300 bp on 2% low-range ultra agarose and then PCR-amplified using Phusion DNA polymerase (NEB, Ipswich, MA, USA) for 15 PCR cycles. The PCR products were purified, and the RNA-Seq libraries were sequenced using an Illumina HiSeq 2500 system (2 × 150 bp read length) at the Shanghai Majorbio Biopharm Technology Co. Ltd. (Shanghai, China). DESeq2 software was used for the statistical analysis of raw counts. The differences in gene expression between the groups were obtained based on certain screening conditions. All the differentially expressed genes (DEGs) were identified by a comparative analysis. A *t*-test (*p* < 0.05) was used to perform Cufflinks (http://sihua.us/Cufflinks.htm) and identify the DEGs in the four treatments (Ck, Fm, Se, and Fm+Se). A Gene Ontology (GO) enrichment analysis, GO annotation, Kyoto Encyclopedia of Genes and Genomes (KEGG) pathway enrichment analysis, and KEGG annotation were also performed. First, the DEG sequences were obtained in the NCBI Nr database and compared using BLAST. The GO annotation information of genes was searched in the GO database using Blast2GO, a functional classification was performed using WEGO software (Ye et al., 2006). The KEGG Orthology results were obtained using KOBAS2.1. The specific biological pathways where a gene or transcript could be involved were obtained by comparison with the KEGG database (Benjamini and Hochberg, 1995; Conesa et al., 2005; Robinson et al., 2010; Grabherr et al., 2011; Xie et al., 2011).

### 2.5 Quantitative real-time reverse transcription PCR for the validation of partial DEGs

Four DEGs were randomly selected for qRT-PCR analysis to verify the validity of RNA-Seq results. Extracted rice total RNA was reverse transcribed using a kit, which was accomplished using a Takara PrimeScript™ RT-PCR Kit (TaKaRa) kit and PCR instrument (EasyCycler, Analytik Jena, Jena, Germany). The *UBC1* gene was used as an internal reference (Ueda et al., 2013). The primers were designed with primer premier 5 (Premier Biosoft International, Palo Alto, CA, USA), the primer sequences are shown in **Table S4**, and qRT-PCR was performed using the CWBiotech UltraSYBR Mixture kit (CWBio, Beijing, China), all procedures were performed according to the kit instructions. **Table S1** showed the qRT-PCR reaction system. An ABI 7500 fluorescence quantitative PCR instrument (Applied Biosystems) was used for quantitative analysis of gene expression (Zhang et al., 2014). Finally, the tendency of gene expression to change in the Fm + Se treatments was compared after qRT-PCR and RNA-Seq analyses to evaluate whether the sequencing results were reliable.

### 2.6 Gene co-expression network construction

A WGCNA was conducted using the WGCNA software package of R (Zhang and Horvath, 2005). The RNA sequencing data was evaluated to remove low-quality genes and samples before the network was constructed. Spearman correlation coefficients were then determined, and a suitable soft threshold was automatically selected. The weighted Spearman result with the ß exponent was converted into an adjacency matrix. The adjacency matrix was then converted into a topological overlap (TOM) matrix to detect patterns of gene expression. Finally, a hierarchical clustering approach was used to construct a gene hierarchy clustering tree, and the comparable modules were then joined. Hub genes were used for strongly linked genes or genes with a high degree of connectivity in the co-expression module. The top 30 genes with the strongest connections were chosen as hub genes, and the genes were analyzed in more detail depending on the size of the module. A network of gene-gene interactions was constructed and visualized using Cytoscape (v. 3.9.1) (Shannon et al., 2003; Langfelder and Horvath, 2008).

### 2.7 Statistical analysis

The data were expressed as an average of three replicates. Microsoft Excel 2016 (Redmond, WA, USA) was used to process the experimental data, which were analyzed using SPSS 20.0 (IBM, Inc., Armonk, NY, USA). Multiple comparisons were performed using a Duncan test at *p*<0.05. A principle coordinates analysis (PCoA) and orthogonal partial least squares discriminant analysis (OPLS-DA) for these ions were analyzed using SIMCA (v.14.1). A *t*-test was used to determine the concentration of characteristic ions of the groups compared. The analytical results were plotted using GraphPad Prism (v.7.00) (La Jolla, CA, USA). A correlation analysis was performed, and the ions were graphed using R software.

## 3 Results

### 3.1 Mycorrhizal colonization and biomass

Compared with the Fm treatment, the treatment of Fm combined with Se did not change the rate of colonization of rice roots (Fm: 29.33 ± 1.15% and Fm+Se: 28.33 ± 5.77%) (**Table 1**), indicating that Se did not affect the growth of Fm in rice roots. In addition, the treatment of Fm combined with Se did not negatively affect the rice biomass (roots and shoots) but instead promoted their growth.

**Table 1:** Effects of arbuscular mycorrhizal fungus (AMF) inoculation and Se fertilization on mycorrhizal colonization and plant biomass.

Groups	Mycorrhizal colonization rates (%)	Biomass (g pot^-1^)
Ck	–	8.79±0.26^b^
Fm	29.33±1.15^a^	9.61±0.04^a^
Se	–	9.21±0.21^b^
Fm+Se	28.33±5.77^a^	9.76±0.14^a^

The different lowercase letters indicate significant differences (n=3, p <no><</no> 0.05) among different treatments. Ck: No inoculation of Fm and additional addition of Se. Fm: Inoculated only with Fm. Se: Only additional Se. Fm+Se: Both were inoculated with Fm and additionally added Se.

### 3.2 Element concentrations in the rice plants

#### 3.2.1 Principal component analysis of the element concentrations in the roots and shoots

The concentrations of macroelements (Mg, P, Ca, and K), micronutrients (Fe, Mn, Ni, Mo, B, Cu, Zn) and beneficial elements (Se, Al, Si, Co) in the shoots and roots were determined to investigate the effect of Fm and Se fertilization on ion metabolism. A principal component analysis (PCA) showed that the ion transport mode of the four groups differed in both the roots (**Figure S1A**) and shoots (**Figure S1B**). Moreover, the application of Fm combined with Se changed the ion metabolism compared with the control. Each group could be distinguished after the roots had been treated. The effect of Fm on ion metabolism was similar to that of the Ck, and they clustered in the first and third quadrants. In addition, the pattern of ion metabolism after the combined application of Se with Fm was similar to that of the Se group. A similar trend was observed in the shoots. Inoculation of the roots with Fm directly affected ion uptake and metabolism in the rice roots, while ion metabolism in the shoots involved complex processes, such as translocation and transformation. As a result, Se can significantly affect the ion metabolism in the shoots more than Fm.

#### 3.2.2 Element concentrations in the roots and shoots

The parameter of OPLS-DA models is shown in **Table S2**. The variable importance in projection (VIP) value measures the importance of ions in metabolic patterns. Higher VIP values indicate that the ions play a key role in changes in the ion metabolic pattern, and such ions are known as characteristic ions (Ren et al., 2020). In this study, the VIP scores were calculated using OPLS-DA (**Figures S2, S3**). The application of Se contributed the most characteristic ions in both the roots and aboveground. When Se was applied alone, the VIP characteristic ions in the roots were Se, Cu, Mo, Ca, and Al (**Figure S3B**), and those in the shoots were Ca, Co, Mg, Se, Ni, and Al (**Figure S4B**). In contrast, when Fm was applied alone, the VIP characteristic ions in the roots were B, Al, Fe, and Ca (**Figure S3A**), and the VIP characteristic ions in the shoots were only Ca and Co (**Figure S4A**). The number of VIP characteristic ions in the roots and aboveground was reversed in the Fm vs Fm+Se and Se vs Fm+Se groups. The VIP feature ions in the roots of the Fm vs Fm+Se group were only Se and Mo (**Figure S3C**), whereas there were four VIP feature ions in the aboveground, including Cu, Al, Mo, and Se (**Figure S4C**). There were five feature ions in the roots of the Se vs Fm+Se group, including Se, Ni, Cu, K, and Mg (**Figure S3D**), while only Se and Ca were present in the aboveground portions of the plant (**Figure S4C**). These results suggest that Fm has a greater effect on the uptake of metal ions in the roots, and the application of Se has a different effect on the uptake of other ions than the effect of translocation.

### 3.3 Transcriptome analysis of the roots

**Figure 1:**
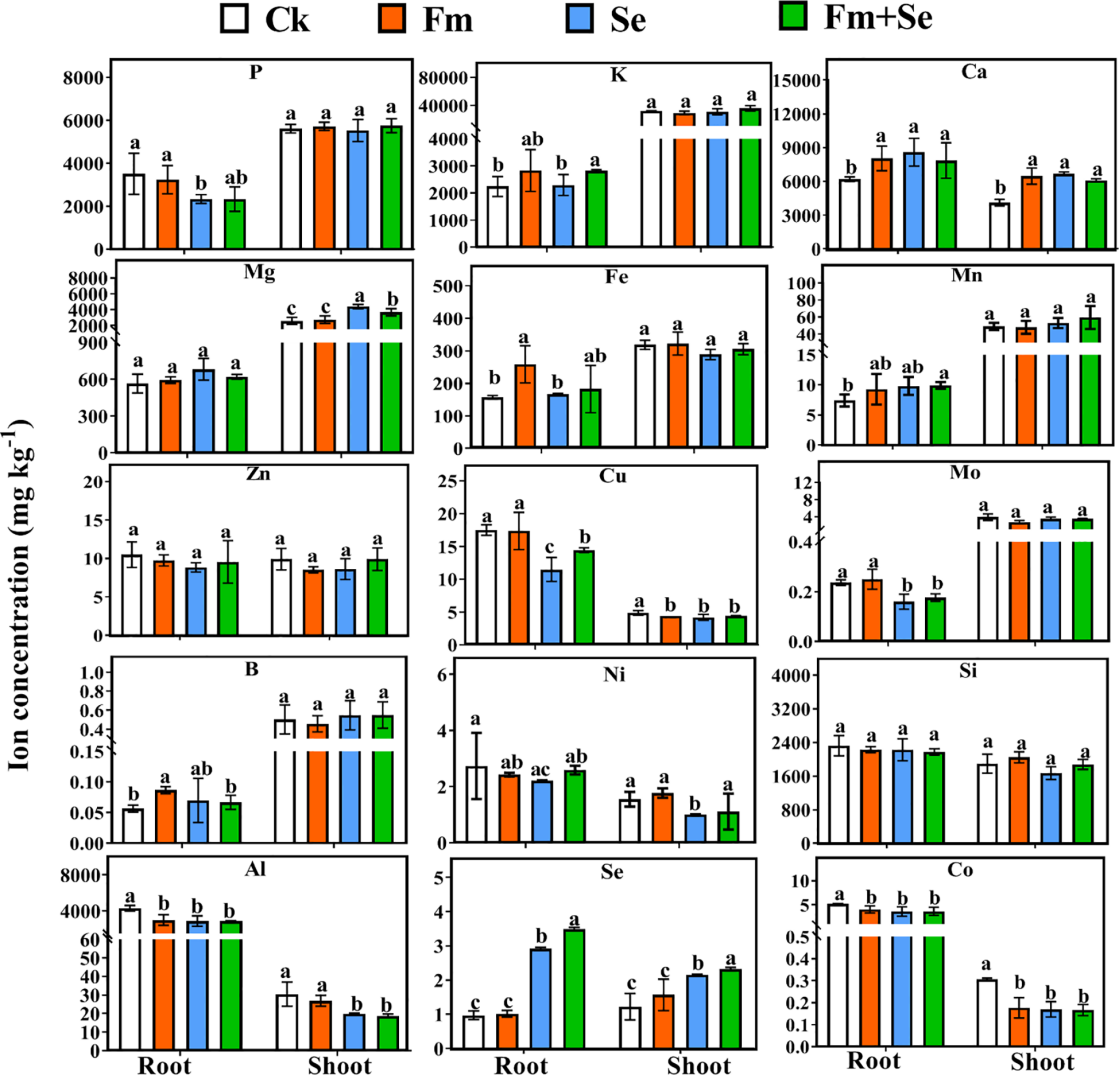
The concentration of ions in the shoots and roots (macronutrients: P, K, Ca and Mg, micronutrients: Fe, Mn, Zn, Cu, Mo, B and Ni, beneficial elements: Si, Al, Se and Co), error bars indicate standard errors of the mean (n = 3), and different letters indicate significant differences (p < 0.05) among different treatments. Ck: No inoculation of Fm nor additional addition of Se. Fm: Inoculated only with Fm. Se: Only additional Se. Fm+Se: Both inoculated with Fm and additionally added Se.

A total of 88.77 Gb clean data with more than 97% that exhibited a quality score of Q20 were obtained after the RNA-Seq of 12 libraries from rice roots. BLASTing was conducted using BLASTx against several public databases, including NR, Swiss-Prot, Pfam, Clusters of Orthologous Genes (COG), GO, and KEGG (E-value < le^-5^) (**Table S3**). **Figure 1**

**Figure 2:**
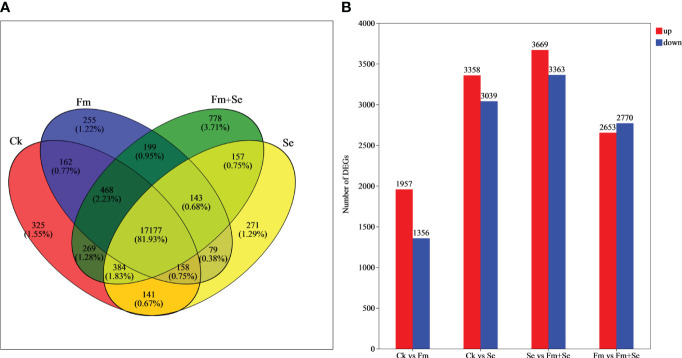
VENN analysis **(A)** and DEGs **(B)** in comparison groups. Ck: No inoculation of Fm nor additional addition of Se. Fm: Inoculated only with Fm. Se: Only additional Se. Fm+Se: Both inoculated with Fm and additionally added Se.

A Venn analysis showed that the number of genes unique to each of the four treatment groups was as follows: 325 in the Ck group, 255 in the Fm group, 271 in the Se group, and 778 in the Fm+Se group (**Figure 2A**). The Ck vs Fm group produced the lowest number of differential genes (3,313), whereas Se vs Fm+Se produced the highest number of differential genes (7,032) (**Figure 2B**).

**Figure 3:**
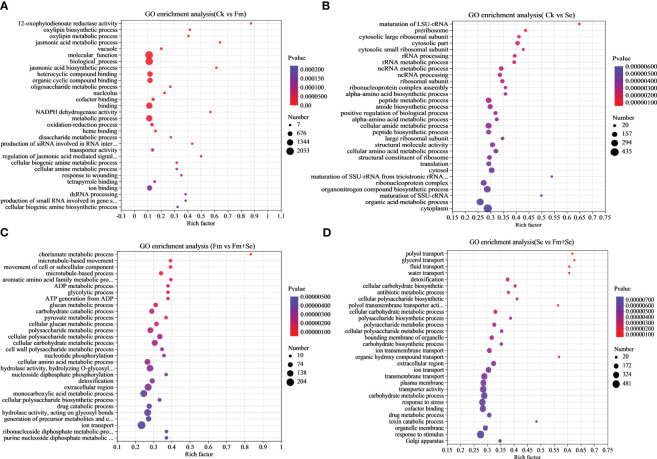
Go enrichment analysis of the DEGs of comparison groups, **(A)** Ck vs Fm, **(B)** Ck vs Se, **(C)** Fm vs Fm+Se, **(D)** Se vs Fm+Se. Ck: No inoculation of Fm nor additional addition of Se. Fm: Inoculated only with Fm. Se: Only additional Se. Fm+Se: Both inoculated with Fm and additionally added Se.

A GO functional enrichment analysis was conducted to identify the GO terms related to the DEGs. The top 30 significantly enriched GO terms are shown in **Figure 3** (*p*<0.05). Most DEGs (2,033) in the Ck vs. Fm groups were related to the “molecular function,” and the GO term related to jasmonic acid metabolism appeared more frequently, including jasmonic acid metabolic processes, jasmonic acid biosynthetic processes, and the regulation of signaling mediated by jasmonic acid (**Figure 3A**), This suggests that colonization with Fm affects the expression of genes related to the synthesis and metabolism of jasmonic acid. Furthermore, 435 DEGs in the Ck vs. Se group were related to “Cytoplasm” (435), and the addition of Se enabled the differential expression of genes associated with rRNA metabolism and also had an effect on alpha-amino acid anabolism (**Figure 3B**). A total of 204 DEGs in the Fm vs. Fm+Se group were related to “ion transport;” several polysaccharide metabolism-related GO terms were enriched, and the addition of Se may have mobilized the genes related to cell wall compared with the application of Fm alone (**Figure 3C**). Notably, some DEGs in both the Fm vs. Fm+Se and Se vs. Fm+Se groups were related to “ion transport.” In addition, some DEGs in the Se vs. Fm+Se group were related to “water transport” and “fluid transport” (**Figure 3D**).

**Figure 4:**
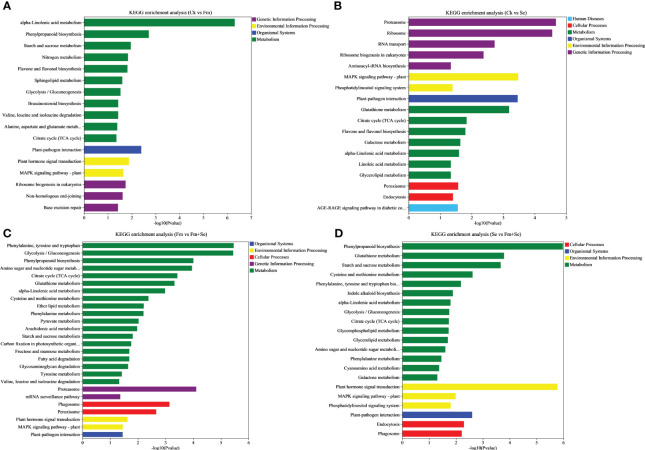
KEGG enrichment analysis of the differentially expressed genes of compare groups, **(A)** Ck vs Fm, **(B)** Ck vs Se, **(C)** Fm vs Fm+Se, **(D)** Se vs Fm+Se. Ck: No inoculation of Fm nor additional addition of Se. Fm: Inoculated only with Fm. Se: Only additional Se. Fm+Se: Both inoculated with Fm and additionally added Se.

A KEGG enrichment analysis was also performed to identify the KEGG terms related to the DEGs. The top 30 significantly enriched KEGG terms are shown in **Figure 4** (*p*<0.05). The DEGs in all the groups were related to “alpha-Linolenic acid metabolism,” “Citrate cycle (TCA cycle),” “Plant-pathogen interaction” and “MAPK signaling pathway.” Furthermore, the DEGS in the Ck vs. Se group (**Figure 4B**), Fm vs. Fm+Se group (**Figure 4C**), and Se vs. Se+Fm group (**Figure 4D**) were related to “Glutathione metabolism.”

**Figure 5:**
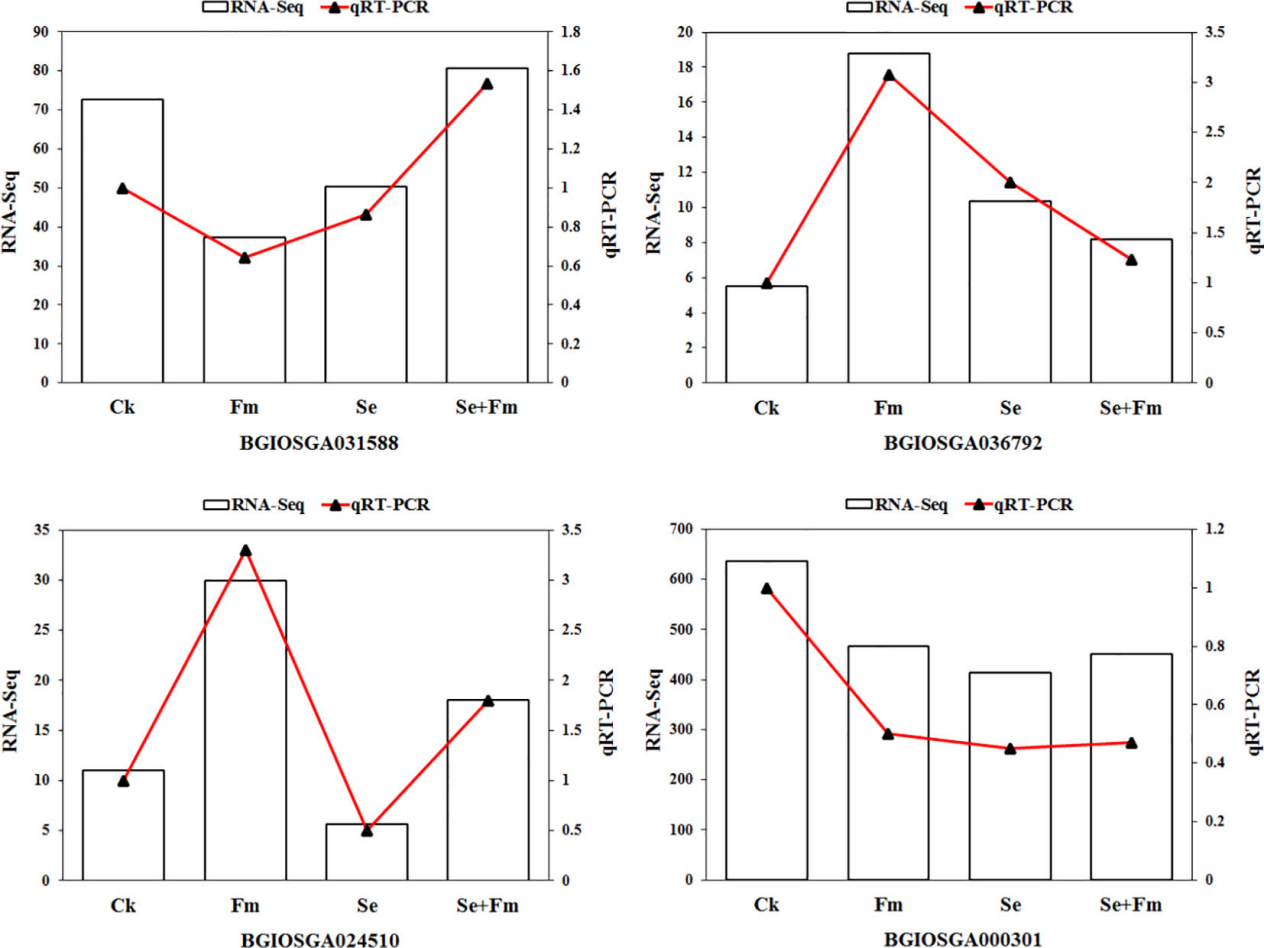
qRT-PCR validation of the expression of randomly selected 4 DEGs that resulted from RNA-Seq.

Four DEGs were randomly selected for qRT-PCR analysis to verify the reliability of the RNA-Seq data (**Figure 5**). The pattern of expression of these genes was consistent with the RNA-Seq results, which suggested that the findings were reliable.

### 3.4 Weighted gene co-expression network analysis (WGCNA)

**Figure 6:**
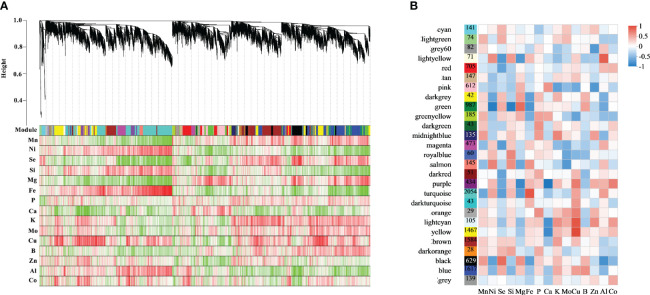
Weighted gene co-expression network of rice roots. **(A)** Clustering dendrogram of genes, with dissimilarity based on the topological overlap, together with assigned module colors. The clustered branches represent different modules, and each line represents one gene. **(B)** Module-trait associations.

A co-expression analysis and network construction (using 12,082 genes) were performed to investigate the gene regulatory network of ion metabolism affected by Fm and Se in the rice roots (**Figure 6**). A total of 27 different modules were identified in a dendrogram. The turquoise module had the most genes (2,054), followed by the blue module (1,617) (**Figure 6B**). The interactions between gene models were determined using a network heatmap for co-expression modules (**Figure 6B**). The hub genes with the highest significant correlation module for the other ions are shown in **Table S5**. Some modules were significantly associated with multiple ions simultaneously, which suggested that this part of the gene may be simultaneously associated with the uptake and accumulation of these ions.

**Figure 7:**
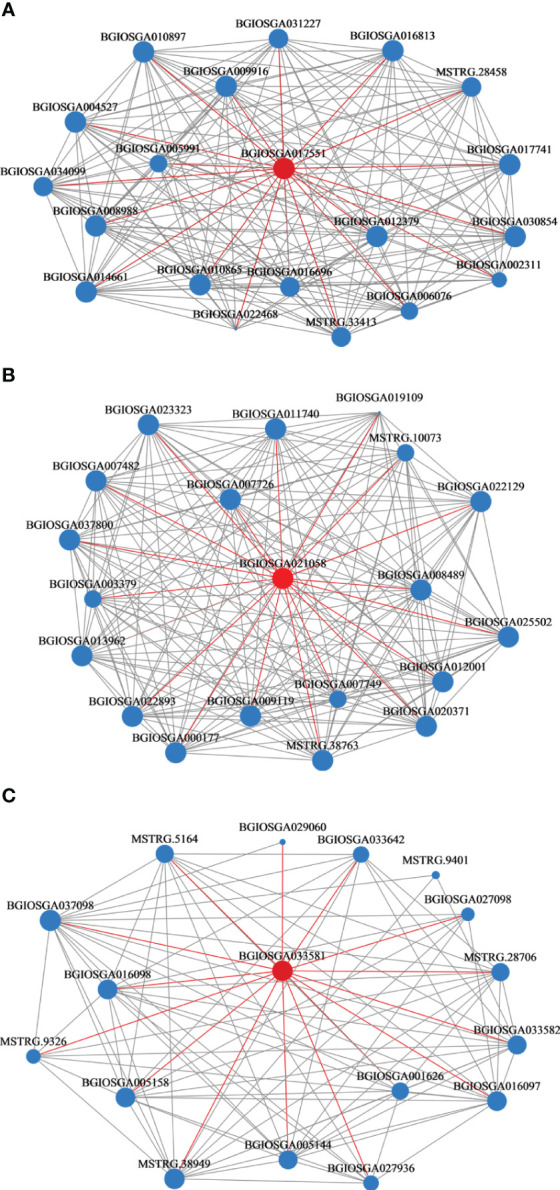
Weighted gene co-expression network of rice roots. **(A)** Black module; **(B)** Light-yellow module; **(C)** Magenta module.

Black modules had the strongest correlation with Se (R=0.71, p<0.05), followed by the light-yellow (R= -0.62, p<0.05) and magenta modules (R= -0.59, p<0.05). Network analyses of 20 hub genes in the black, light-yellow, and magenta modules are shown in **Figure 7**. *BGIOSGA017551* (black), *BGIOSGA033581* (light-yellow), and *BGIOSGA021058* (magenta) had the highest degree of correlation in the modules. *BGIOSGA017551* was related to ATP binding, transferase activity, protein serine/threonine kinase activity, MAPK cascade, and protein phosphorylation, while *BGIOSGA033581* was annotated to protein phosphorylation, cell surface receptor signaling pathway, protein serine/threonine kinase activity, protein kinase activity and ATP binding. Although there was no relevant GO annotation information for *BGIOSGA021058*, its homolog in *O. sativa* Japonica, *Os06g0552400*, was annotated to ruffle membrane, metal ion binding, phosphatidylinositol binding and regulation of ruffle assembly (**Tables S6-8**). This could be related to the transport of metal ions through the plant cell membranes.

## 4 Discussion

### 4.1 Effect of Fm inoculation and Se application on the rice growth and Se uptake

Previous studies have shown that AMF readily establishes symbiotic relationships with many plant species, which facilitates the uptake of nutrients and water by plants. Thus, the application of AMF benefits the growth of plants by improving crop nutrition and stress resistance in particular and tolerance to increase the yields (Begum et al., 2019; Jabborova et al., 2022). Researchers have proven that the presence of AMF improves the utilization of beneficial elements in the soil and that these beneficial elements are transferred by the symbiont hyphae to the host roots (Kaya et al., 2009; Subramanian et al., 2013; Chen et al., 2017). Moreover, transcriptome analyses showed that the DEGs of Ck vs Fm were significantly enriched in several Go terms related to jasmonic acid, and the KEGG term of flavone biosynthesis was enriched. This indicated that rice had become more resilient to stress, which is similar to the findings of previous studies (He et al., 2017; Lu et al., 2020). This suggests that inoculation with Fm promotes the growth of rice, which is also reflected in the biomass.

The application of Se also increased the rice biomass in this study, while the transcriptome analysis showed that the DEGs were enriched in the glutathione metabolic pathway and flavonoid biosynthesis pathway. This is consistent with previous findings that Se can stimulate the production of secondary metabolites in plants and increase their tolerance to stress, which affects the uptake of Se by the roots (Feng et al., 2021; Huang et al., 2023). Sun et al. (2021) reported that the fungal colonization rates of Fm in maize (*Zea mays* L. cv. Guidan 0810) steadily increased when increasing levels of Se were applied, peaked at the treatment level of 5 mg kg^-1^ Se, and then began to decrease as the levels of Se applied to the soil increased to 10 mg kg^-1^ Se. The dose of Se applied in this experiment did not affect the colonization by Fm in rice. This implies that the colonization of Fm in crops is facilitated by the application of Se at doses that are within reasonable limits. This provides a reference for the combined use of Fm fungal and Se fertilizers in crop cultivation.

The combination of AMF inoculation with the application of Se increased the biomass of wheat (*Triticum aestivum* L. cv. Xiaoyan 22), rice (*Oryza sativa* L. cv. Xinliangyou 6), and soybean (*Glycine max* (Linn.) Merr. Meilifengmao NO. 2), and inoculation with AMF also increased the Se concentration of the grains of these crops (Chen et al., 2020; Li et al., 2022; Zhang et al., 2022), which were obtained the similarity in this study. Some studies have found that inoculation with AMF promotes root growth, which could be one of the reasons for the promotion of Se uptake in plants (Zhang et al., 2022). In addition, inoculation with AMF can increase the concentration of available Se in the soil, such as by increasing the proportion of Se in the exchangeable and water-soluble forms in the soil (Dinh et al., 2017; Li et al., 2022; Guo et al., 2023). Peng et al. (2020) reported that the synergistic effect of colonization by AMF and low P input promoted the uptake of Se by alfalfa (*Medicago sativa* L. cv Golden Empress). However, the mechanisms of how AMF promotes Se uptake in plants are still not fully understood and may vary depending on the specific conditions and plant species. Further research is needed to elucidate the exact mechanisms involved in this process. Overall, appropriate doses of Se application and AMF inoculation can promote plant growth and Se concentration, and Fm can act as a crop-friendly bioenhancer for Se fertilization.

### 4.2 Effects of Fm inoculation and Se application on ionomic responses in rice seedlings

In this study, the concentrations of metals in the roots and shoots of rice were determined. K, Ca, Mg, P are essential macronutrients in rice plants, Fe, Mn, Zn, Mo, B, Cu, Ni are micronutrients which plays important role in plant growth, Se, Al, Co and Si are the beneficial elements which stimulate plants growth (Arif et al., 2016; Kumar et al., 2017; Shrestha et al., 2020; Imran et al., 2021; Ramírez-Olvera et al., 2021; Rengel et al., 2022). Therefore, this experiment examined the effects of inoculation with Fm and application of Se on the concentrations of these elements in rice plants. Inoculation with Fm increased the concentrations of K, Ca, Mg in the rice roots, and Ca was the vip ions of roots in Ck vs Fm group, this is consistent with previous research. Yuan et al. (2023) found that AMF (*Funneliformis mosseae* BGC XJ02 and *Claroideoglomus etunicatum* BGC XJ03C) improved plant K nutrition, but the improvement was related to the species of AMF, Carrara and Heller (2022) found that AMF(*Claroideoglumus etunicatum* PA127A and *Gigaspora margarita* NC175) increased Ca and Mg concentration in corn (*Zea mays* cv.Coastal (Seedway, Hall NY)). It may be that the inoculation of AMF improves the ability of plants to cope with stress and thus absorb more nutrients (Chandrasekaran et al., 2021). B, Fe and Al, also were the VIP ions in the roots, and Co were the VIP ions in shoots in the Ck vs Fm group. B is crucial for the higher growth of plants and development in the transport of sugars, hormones, and phenolic compounds, and inoculation with Fm ameliorates the adverse effects of B deficiency on tea trees and improves the uptake of B by tea oil camellia (*Camellia oleifera* Abel. cv. Chang Lin 40) seedlings (Liu et al., 2023). AMF have also been shown to increase the availability of Fe in the soil by stimulating the secretion of phenolic acids from the roots of quince (*Cydonia oblonga* Mill. cv. Isfahan) to enhance the uptake of Fe (Rahimi et al., 2021), and, in turn, alleviate abiotic stress in plants. In contrast, inoculation with AMF had a limiting effect on the uptake of Al in rice, and inoculation with AMF could provide new mechanisms for the exclusion and detoxification of Al, such as metal immobilization in extra- and intraradical mycelia (Alotaibi et al., 2021). Moreover, AMF regulates the expression of host genes related to metal transport (Luo et al., 2022). Overall, inoculation with AMF affects the uptake of elements from the soil by rice.

The application of Se also had an effect on the uptake and accumulation of ions in rice, but it was somewhat different from that of AMF. The application of Se in this experiment reduced the concentration of P in rice roots. Previous research has demonstrated that Se and P interact antagonistically at the site of plant uptake, which decreases the rate of Se uptake by plant roots (Jiang et al., 2022). Se can promote the expression of Mn transporters (ZRT/IRT family member [IRT1]), thus, promoting the uptake of N in *Brassica chinensis* L. (Wang et al., 2022) and significantly increasing the accumulation of Mn in olive trees (*Olea europaea* L. cv. Leccino) (D’Amato et al., 2018). Similarly, the application of Se in this study also reduced the concentration of Mo in rice roots, which is similar to the findings of previous studies. The effect of Se on the uptake of Mo by plants was also related to the concentrations of Mo and Se of the culture additions, with the application of Se decreasing the concentration of Mo in the roots of Chinese cabbage (*Brassica campestris* L. ssp. Pekinensis) at a Mo concentration of 0.01-0.1 mg L^-1^ (Zhang et al., 2012). Se is absorbed by plant roots via the phosphate transporter and thus, induces an antagonistic effect with Mo and P (Zhang et al., 2012; Zafeiriou et al., 2022). Landberg and Greger (1994) found that Se depressed the uptake of Cu in pea (*Pisum sativum* L. cv. Fenomen) but had no significant effect on wheat (*Triticum aestivum* L. cv. Sunny), and the application of Se to rice reduced its uptake and accumulation of Cu. However, the mechanism of the effect of Se on Cu uptake in plants has not yet been studied, and more experiments are needed for an in-depth exploration of this mechanism. Plants only require very small concentrations of Ni (Arif et al., 2016). Other studies found that the effect of Se on Ni uptake depends on the concentration and form of Se used and that Se decreased the concentration of Ni in lettuce (*Lactuca sativa* var. *capitata* L. cv. Justynaroots) but increased its concentration in the shoots (Hawrylak-Nowak and Matraszek-Gawron, 2020). Se also reduced the concentration of Al in rice roots and shoots. Studies on ryegrass (Lolium perenne L. cv. Nui) suggest that Se may respond to Al stress by increasing the plant’s antioxidant capacity, thereby reducing the accumulation of Al (Cartes et al., 2010). However, there was also studied that Se does not ease growth inhibition caused by Al in *Schinus terebinthifolius* seedlings (De Mattos et al., 2023). These results contrast with those of this study, and in-depth studies on the interplay between Se and Ni or Al in plants are still required.

Although the effects of Se or Fm alone on the uptake of metal ions by rice were not identical, some of the inhibitory effects exhibited by Se were reduced when they were applied together, probably because Fm regulates the physicochemical properties of the soil and promotes nutrient uptake by the roots (Jabborova et al., 2022; Ma et al., 2022b). However, there was a consistent effect of reducing the absorption of Al. Treatment with Se and Fm can reduce the accumulation of Al in plants (Rufyikiri et al., 2008; Cartes et al., 2010). Although no study has reported the interaction between Co with Se and Fm in plants, studies have suggested that the metabolic pattern of Co in plants is similar to that of Ni (Hu et al., 2021). In summary, Fm and Se affect the uptake of ionomic by rice seedlings.

### 4.3 The genes related to metal ion uptake in rice following the application of Fm and Se

**Table S5** shows the modules significantly associated with ions and the hub genes of the highest degree in modules. *BGIOSGA033581* was a hub gene in the light-yellow module, which significantly correlated with Se and Mg and significantly positively correlated with Al. The homologue of *BGIOSGA033581* is *Os11g0667700* (OsRLCK348), and *BGIOSGA034707* is another hub gene in the light-yellow module, which also represents the *RLCK* gene (**Table S7**). RLCK (Receptor-Like Cytoplasmic Kinase) genes are a family of genes in rice that belong to the superfamily of receptor-like kinases (RLKs). Some of the functionally characterized RLCKs from plants have been shown to play roles in development and stress responses (Vij et al., 2008). RLCK348 could be related to the resistance of rice to salt stress (Patishtan et al., 2018) and the possible association of RLCK with biostimulation (Lehti-Shiu et al., 2009). However, there is no direct information on the role of RLCK genes in the uptake of metal ions by rice. Further research may be needed to determine the specific effects of RLCK genes on the uptake of metal ions by rice.

Previous studies have suggested that the uptake of ions by plants is influenced by genes associated with the cell membrane (Jain et al., 2018). In this study, the hub genes related to the binding of metal ions (*BGIOSGA010865, BGIOSGA016813, BGIOSGA005991, BGIOSGA037098, BGIOSGA025502*, and *BGIOSGA008466*) and a zinc finger protein-related gene (*BGIOSGA007726*) were identified. Genes related to membrane transport also appeared in the hub genes analyzed by WGCNA, including *BGIOSGA008466, BGIOSGA010865, BGIOSGA004527, BGIOSGA017741, BGIOSGA005144, BGIOSGA029060*, and *BGIOSGA037800*. The cell wall also plays a role in the responses of plant cells to trace metals (Krzesłowska, 2011), and genes concerning the cell wall have also been localized to hub genes (*BGIOSGA017741*). These genes may also have been affected by inoculation with Fm. Studies have shown that AMF can affect the expression of genes related to the plasma membrane and cell wall activities, and Fm can positively affect plant cellular components, biological process, and molecular function, and the expression of genes related to the transport of metal ion transports in plants (Lu et al., 2020; Moradi et al., 2020; Luo et al., 2022). Therefore, the application of Fm combined with Se can affect the expression of genes related to the uptake of metal ions and Se metabolism (glutathione metabolism). In addition, the hub gene *BGIOSGA017551* was annotated to the MAPK cascade, which is a pathway associated with the response to Se (Rashtchizadeh et al., 2015). *BGIOSGA017551, BGIOSGA004527, BGIOSGA009916, BGIOSGA033581*, and *BGIOSGA011740* were annotated to phosphorylation. However, the relationship between the absorption of Se with phosphorylation and ATP binding in plants is unknown. Nevertheless, studies have shown that Se can regulate photosynthesis in plants (Liu et al., 2022). Photophosphorylation is the process by which ADP is phosphorylated to form ATP using sunlight. Simultaneously, six other hub genes were annotated to protein serine/threonine kinase activity (*BGIOSGA033581, BGIOSGA017551, BGIOSGA004527, BGIOSGA009916, BGIOSGA033581*, and *BGIOSGA011740*). Studies have shown that Se is also involved in the synthesis of cysteine owing to a protein-protein interaction with serine (Trippe and Pilon-Smits, 2021), which indicates that these hub genes are related to Se. In summary, the simultaneous application of Fm and Se may affect the expression of the related genes of transporters, metal ion binding phosphorylation, and protein serine/threonine kinase activity in the cell membrane of rice roots.

## 5 Conclusions

Fm fungi can significantly promote the growth of rice. Furthermore, these fungi can form a mutually beneficial symbiotic relationship and promote the absorption and transport of Se in rice. However, the application of Fm combined with Se is more effective at promoting these effects than that of Se alone. The combined application of Fm and Se can also influence ion metabolism in rice since Fm reduces the antagonism of Se on the absorption of Ni, Mo, P, and Cu. Herein, a WGCNA analysis showed that some genes were associated with membrane, phosphorylation, and metal ion binding, indicating that Fm affects the expression of these genes and the uptake of Se by roots. Overall, Fm can be used as a bioenhancer to produce fertilizers enriched in SE during the production of crops enriched in this element.

## Data availability statement

The datasets presented in this study can be found in online repositories. The names of the repository/repositories and accession number(s) can be found below: BioProject, PRJNA988279.

## Author contributions

YQ: conceptualization, methodology, formal analysis, and writing. QC: conceptualization, methodology, formal analysis, and writing. YL: conceptualization, field sampling, analysis and writing. XC: methodology and formal analysis. JX: field sampling and analysis. GH: Formal analysis, and writing. XY: writing and revision. SL: writing and revision, XMY: field sampling and analysis, DL: writing and revision, XW: writing and revision. YW, methodology, formal analysis, writing, and funding acquisition. All authors contributed to the article and approved the submitted version.

## References

[B1] Alotaibi M. O. Saleh A. M. Sobrinho R. L. Sheteiwy M. S. El-Sawah A. M. Mohammed A. E.. (2021). Arbuscular mycorrhizae mitigate aluminum toxicity and regulate proline metabolism in plants grown in acidic soil. J. Fungi. 7 (7), 531. doi: 10.3390/jof7070531 PMC8304902 34209315

[B2] Arif N. Yadav V. Singh S. Singh S. Ahmad P. Mishra R. K.. (2016). Influence of high and low levels of plant-beneficial heavy metal ions on plant growth and development. Front. Env. Sci-Switz. 4. doi: 10.3389/fenvs.2016.00069

[B3] Bamberg S. M. Ramos S. J. Carbone M. A. C. Siqueira J. O. (2019). Effects of selenium (Se) application and arbuscular mycorrhizal (AMF) inoculation on soybean (‘Glycine max’) and forage grass (‘Urochloa decumbens’) development in oxisol. Aust. J. Crop Sci. 13 (3), 380–385. doi: 10.21475/ajcs.19.13.03.p1245

[B4] Begum N. Qin C. Ahanger M. A. Raza S. Khan M. I. Ashraf M.. (2019). Role of arbuscular mycorrhizal fungi in plant growth regulation: implications in abiotic stress tolerance. Front. Plant Sci. 10. doi: 10.3389/fpls.2019.01068 PMC6761482 31608075

[B5] Benjamini Y. Hochberg Y. (1995). Controlling the false discovery rate: a practical and powerful approach to multiple testing. J. R Statist Soc 57, 289–300. doi: 10.1111/j.2517-6161.1995.tb02031.x

[B6] Bernaola L. Cange G. Way M. Gore J. Hardke J. Stout M. (2018). Natural colonization of rice by arbuscular mycorrhizal fungi in different production areas. Rice Sci. 25 (3), 169–174. doi: 10.1016/j.rsci.2018.02.006

[B7] Campo S. Martín-Cardoso H. Olivé M. Pla E. Catala-Forner M. Martínez-Eixarch M.. (2020). Effect of root colonization by arbuscular mycorrhizal fungi on growth, productivity and blast resistance in rice. Rice 13 (1), 1–14. doi: 10.1186/s12284-020-00402-7 32572623 PMC7310045

[B8] Carrara J. E. Heller W. P. (2022). Arbuscular mycorrhizal species vary in their impact on nutrient uptake in sweet corn (Zea mays) and butternut squash (Cucurbita moschata). Front. Agron. 4. doi: 10.3389/fagro.2022.1040054

[B9] Cartes P. Jara A. A. Pinilla L. Rosas A. Mora M. L. (2010). Selenium improves the antioxidant ability against aluminium-induced oxidative stress in ryegrass roots. Ann. Appl. Biol. 156 (2), 297–307. doi: 10.1111/j.1744-7348.2010.00387.x

[B10] Chandrasekaran M. Boopathi T. Manivannan P. (2021). Comprehensive assessment of ameliorative effects of AMF in alleviating abiotic stress in tomato plants. J. Fungi. 7 (4), 303. doi: 10.3390/jof7040303 PMC8071382 33921098

[B11] Chen M. Yang G. Sheng Y. Li P. Qiu H. Zhou X.. (2017). *Glomus mosseae* inoculation improves the root system architecture, photosynthetic efficiency and flavonoids accumulation of liquorice under nutrient stress. Front. Plant Sci. 8. doi: 10.3389/fpls.2017.00931 PMC5461296 28638391

[B12] Chen X. Zhang Z. Gu M. H. Li H. Shohag M. J. I. Shen F. K.. (2020). Combined use of arbuscular mycorrhizal fungus and selenium fertilizer shapes microbial community structure and enhances organic selenium accumulation in rice grain. Sci. Total Environ. 748, 141166. doi: 10.1016/j.scitotenv.2020.141166 32798860

[B13] Conesa A. Götz S. García-Gómez J. M. Terol J. Talón M. Robles M. (2005). Blast2GO: a universal tool for annotation, visualization and analysis in functional genomics research. Bioinformatics. 21 (18), 3674–3676. doi: 10.1093/bioinformatics/bti610 16081474

[B14] D’Amato R. Petrelli M. Proietti P. Onofri A. Regni L. Perugini D.. (2018). Determination of changes in the concentration and distribution of elements within olive drupes (cv. Leccino) from Se biofortified plants, using laser ablation inductively coupled plasma mass spectrometry. J. Sci. Food. 98 (13), 4971–4977. doi: 10.1002/jsfa.9030 29577309

[B15] D’Amato R. Regni L. Falcinelli B. Mattioli S. Benincasa P. Dal Bosco A.. (2020). Current knowledge on selenium biofortification to improve the nutraceutical profile of food: a comprehensive review. J. Agri.c Food Chem. 68 (14), 4075–4097. doi: 10.1021/acs.jafc.0c00172 PMC7997367 32181658

[B16] De Mattos J. P. O. Aguilar M. V. M. Alves J. D. S. Birck T. P. Kuinchtner C. C. Tarouco C. P.. (2023). Selenium does not ease growth inhibition caused by aluminum in Schinus terebinthifolius seedlings. J. Plant Nutr. 46 (14), 1–18. doi: 10.1080/01904167.2023.2206427

[B17] Dinh Q. T. Cui Z. W. Huang J. Tran T. A. T. Wang D. Yang W.. (2018). Selenium distribution in the Chinese environment and its relationship with human health: A review. Environ. Int. 112, 294–309. doi: 10.1016/j.envint.2017.12.035 29438838

[B18] Dinh Q. T. Li Z. Tran T. A. T. Wang D. Liang D. (2017). Role of organic acids on the bioavailability of selenium in soil: A review. Chemosphere. 184, 618–635. doi: 10.1016/j.chemosphere.2017.06.034 28624740

[B19] Feng Z. W. Sun H. Qin Y. Q. Zhou Y. Zhu H. H. Yao Q. (2023). A synthetic community of siderophore-producing bacteria increases soil selenium bioavailability and plant uptake through regulation of the soil microbiome. Sci. Total Environ. 871, 162076. doi: 10.1016/j.scitotenv.2023.162076 36758687

[B20] Feng R. W. Wang L. Z. Yang J. G. Zhao P. P. Zhu M. Y. Li Y. P.. (2021). Underlying mechanisms responsible for restriction of uptake and translocation of heavy metals (metalloids) by selenium via root application in plants. J. Hazard Mater. 402, 123570. doi: 10.1016/j.jhazmat.2020.123570 32745877

[B21] Grabherr M. G. Haas B. J. Yassour M. Levin J. Z. Thompson D. A. Amit I.. (2011). Trinity: reconstructing a full-length transcriptome without a genome from RNA-Seq data. Nat. Biotechnol. 29 (7), 644. doi: 10.1038/nbt.1883 21572440 PMC3571712

[B22] Griffiths M. York L. M. (2020). Targeting root ion uptake kinetics to increase plant productivity and nutrient use efficiency. Plant Physiol. 182 (4), 1854–1868. doi: 10.1104/pp.19.01496 32029523 PMC7140967

[B23] Gui J. Y. Rao S. Huang X. R. Liu X. M. Cheng S. Y. Xu F. (2022). Interaction between selenium and essential micronutrient elements in plants: A systematic review. Sci. Total Environ. 853, 158673. doi: 10.1016/j.scitotenv.2022.158673 36096215

[B24] Guo Q. X. Ye J. H. Zeng J. M. Chen L. Korpelainen H. Li C. Y. (2023). Selenium species transforming along soil-plant continuum and their beneficial roles for horticultural crops. Hortic. Res. 10 (2), uhac270. doi: 10.1093/hr/uhac270 36789256 PMC9923214

[B25] Gupta M. Gupta S. (2016). An overview of selenium uptake, metabolism, and toxicity in plants. Front. Plant Sci. 7. doi: 10.1016/j.scitotenv.2023.162076 PMC5225104 28123395

[B26] Hasanuzzaman M. Bhuyan M. B. Raza A. Hawrylak-Nowak B. Matraszek-Gawron R. Nahar K.. (2020). Selenium toxicity in plants and environment: biogeochemistry and remediation possibilities. Plants 9 (12), 1711. doi: 10.3390/plants9121711 33291816 PMC7762096

[B27] Hawrylak-Nowak B. Matraszek-Gawron R. (2020). Difference between selenite and selenate in the regulation of growth and physiological parameters of nickel-exposed lettuce. Biology. 9 (12), 465. doi: 10.3390/biology9120465 33322708 PMC7763836

[B28] He L. Li C. Liu R. (2017). Indirect interactions between arbuscular mycorrhizal fungi and *Spodoptera exigua* alter photosynthesis and plant endogenous hormones. Mycorrhiza. 27, 525–535. doi: 10.1007/s00572-017-0771-2 28424944

[B29] Hu X. Wei X. Y. Ling J. Chen J. J. (2021). Cobalt: an essential micronutrient for plant growth? Front. Plant Sci. 12. doi: 10.3389/fpls.2021.768523 PMC8635114 34868165

[B30] Huang J. Q. Wang Z. H. Sun L. H. Wang L. L. Yin Y. L. (2023). Selenium in modern agriculture. Modern Agriculture. 1 (1), 34–42. doi: 10.1002/moda.2

[B31] Imran M. Hussain S. Rana M. S. Saleem M. H. Rasul F. Ali K. H.. (2021). Molybdenum improves 2-acetyl-1-pyrroline, grain quality traits and yield attributes in fragrant rice through efficient nitrogen assimilation under cadmium toxicity. Ecotoxicol. Environ. Safety. 211, 111911. doi: 10.1016/j.ecoenv.2021.111911 33453638

[B32] Jabborova D. Davranov K. Jabbarov Z. Bhowmik S. N. Ercisli S. Danish S.. (2022). Dual inoculation of plant growth-promoting *Bacillus endophyticus* and *Funneliformis mosseae* improves plant growth and soil properties in ginger. ACS Omega. 7 (39), 34779–34788. doi: 10.1021/acsomega.2c02353 36211029 PMC9535732

[B33] Jain S. Muneer S. Guerriero G. Liu S. Vishwakarma K. Chauhan D. K. (2018). Tracing the role of plant proteins in the response to metal toxicity: a comprehensive review. Plant Signal Behav. 13 (9), e1507401. doi: 10.1080/15592324.2018.1507401 30188762 PMC6204846

[B34] Jiang Y. N. Wang W. X. Xie Q. J. Liu N. Liu L. X. Wang D. P.. (2017). Plants transfer lipids to sustain colonization by mutualistic mycorrhizal and parasitic fungi. Science. 356 (6343), 1172–1175. doi: 10.1126/science.aam9970 28596307

[B35] Jiang T. Yu T. Qi H. Li F. Yang Z. (2022). Analysis of phosphorus and sulfur effect on soil selenium bioavailability based on diffusive gradients in thin films technique and sequential extraction. Chemosphere. 302, 134831. doi: 10.1016/j.chemosphere.2022.134831 35523297

[B36] Kabir A. H. Debnath T. Das U. Prity S. A. Haque A. Rahman M. M.. (2020). Arbuscular mycorrhizal fungi alleviate Fe-deficiency symptoms in sunflower by increasing iron uptake and its availability along with antioxidant defense. Plant Physiol. Biochem. 150, 254–262. doi: 10.1016/j.plaphy.2020.03.010 32171164

[B37] Kaya M. Kucukyumuk Z. Erdal I. (2009). Phytase activity, phytic acid, zinc, phosphorous and protein contents in different chickpea genotypes in relation to nitrogen and zinc fertilization. Afr. J. Biotechnol. 15, 4508–4513. doi: 10.5897/AJB09.983

[B38] Khan Y. Shah S. Hui T. (2022). The roles of arbuscular mycorrhizal fungi in influencing plant nutrients, photosynthesis, and metabolites of cereal crops—a review. Agronomy. 12 (9), 2191. doi: 10.3390/agronomy12092191

[B39] Krzesłowska M. (2011). The cell wall in plant cell response to trace metals: polysaccharide remodeling and its role in defense strategy. Acta Physiol. Plan. 33, 35–51. doi: 10.1007/s11738-010-0581-z

[B40] Kumar A. Sen A. Upadhyay P. K. Singh R. K. (2017). Effect of zinc, iron and manganese levels on quality, micro and macro nutrients content of rice and their relationship with yield. Commun. Soil Sci. Plan. 48 (13), 1539–1551. doi: 10.1080/00103624.2017.1373799

[B41] Landberg T. Greger M. (1994). Influence of selenium on uptake and toxicity of copper and cadmium in pea (*Pisum sativum*) and wheat (*Triticum aestivum*). Physiol. Plantarum. 90 (4), 637–644. doi: 10.1111/j.1399-3054.1994.tb02518.x

[B42] Langfelder P. Horvath S. (2008). WGCNA: an R package for weighted correlation network analysis. BMC Bioinf. 9 (1), 1–13. doi: 10.1186/1471-2105-9-559 PMC2631488 19114008

[B43] Lehti-Shiu M. D. Zou C. Hanada K. Shiu S. H. (2009). Evolutionary history and stress regulation of plant receptor-like kinase/pelle genes. Plant Physiol. 150 (1), 12–26. doi: 10.1104/pp.108.134353 19321712 PMC2675737

[B44] Lemly A. D. (1997). Environmental implications of excessive selenium: a review. BioMed. Environ. Sci. 10 (4), 415–435. doi: 10.1016/S0147-6513(03)00095-2 9448924

[B45] Li J. Liu R. F. Wu B. Y. Zhang C. Y. Wang J. F. Lyu L. H.. (2022). Influence of arbuscular mycorrhizal fungi on selenium uptake by winter wheat depends on the level of selenate spiked in soil. Chemosphere. 291, 132813. doi: 10.1016/j.chemosphere.2021.132813 34752832

[B46] Li H. Ye Z. H. Chan W. F. Chen X. W. Wu F. Y. Wu S. C.. (2011). Can arbuscular mycorrhizal fungi improve grain yield, As uptake and tolerance of rice grown under aerobic conditions? Environ. pollut. 159 (10), 2537–2545. doi: 10.1016/j.envpol.2011.06.017 21737190

[B47] Lima L. W. Pilon-Smits E. A. H. Schiavon M. (2018). Mechanisms of selenium hyperaccumulation in plants: A survey of molecular, biochemical and ecological cues. Bba-Gen Subjects. 1862 (11), 2343–2353. doi: 10.1016/j.bbagen.2018.03.028 29626605

[B48] Liu H. D. Xiao C. M. Qiu T. C. Deng J. Cheng H. Cong X.. (2022). Selenium regulates antioxidant, photosynthesis, and cell permeability in plants under various abiotic stresses: a review. Plants (Basel). 12 (1), 44. doi: 10.3390/plants12010044 36616173 PMC9824017

[B49] Liu J. Zhang M. Fan J. Ding W. Chen L. Luo J.. (2023). The synergistic effects of AMF inoculation and boron deficiency on the growth and physiology of *camellia oleifera* seedlings. Forests. 14 (6), 1126. doi: 10.3390/f14061126

[B50] Lu C. C. Guo N. Yang C. Sun H. B. Cai B. Y. (2020). Transcriptome and metabolite profiling reveals the effects of *Funneliformis mosseae* on the roots of continuously cropped soybeans. BMC Plant Biol. 20 (1), 479. doi: 10.1186/s12870-020-02647-2 33087042 PMC7579952

[B51] Luginbuehl L. H. Menard G. N. Kurup S. Erp H. Radhakrishnan G. V. Breakspear A.. (2017). Fatty acids in arbuscular mycorrhizal fungi are synthesized by the host plant. Science. 356 (6343), 1175–1178. doi: 10.1126/science.aan0081 28596311

[B52] Luo J. Yan Q. X. Yang G. Wang Y. (2022). Impact of the arbuscular mycorrhizal fungus *Funneliformis mosseae* on the physiological and defence responses of *Canna indica* to copper oxide nanoparticles stress. J. Fungi. 8 (5), 513. doi: 10.3390/jof8050513 PMC9146287 35628768

[B53] Lyu C. H. Chen J. W. Li L. Zhao Z. Q. Liu X. W. (2022). Characteristics of Se in water-soil-plant system and threshold of soil Se in seleniferous areas in Enshi, China. Sci. Total Environ. 827, 154372. doi: 10.1016/j.scitotenv.2022.154372 35259387

[B54] Ma J. Wang W. Yang J. Qin S. Yang Y. Sun C.. (2022b). Mycorrhizal symbiosis promotes the nutrient content accumulation and affects the root exudates in maize. BMC Plant Biol. 22 (1), 64. doi: 10.1186/s12870-021-03370-2 35123400 PMC8817564

[B55] Ma S. L. Zhu L. J. Wang J. P. Liu X. Jia Z. H. Li C.. (2022a). Arbuscular mycorrhizal fungi promote *Gleditsia sinensis* Lam. root growth under salt stress by regulating nutrient uptake and physiology. Forests. 13 (5), 688. doi: 10.3390/f13050688

[B56] Moradi T. Iranbakhsh A. Mehregan I. Ahmadvand R. (2020). Impact of arbuscular mycorrhizal fungi (AMF) on gene expression of some cell wall and membrane elements of wheat (*Triticum aestivum* L.) under water deficit using transcriptome analysis. Physiol. Mol. Biol. Plants. 26, 143–162. doi: 10.1007/s12298-019-00727-8 32153322 PMC7036378

[B57] Patishtan J. Hartley T. N. Fonseca de Carvalho R. Maathuis F. J. (2018). Genome-wide association studies to identify rice salt-tolerance markers. Plant Cell Environ. 41 (5), 970–982. doi: 10.1111/pce.12975 28436093

[B58] Peng Q. Wu M. Zhang Z. Su R. He H. Zhang X. (2020). The interaction of arbuscular mycorrhizal fungi and phosphorus inputs on selenium uptake by alfalfa (*Medicago sativa* L.) and selenium fraction transformation in soil. Front. Plant Sci. 11. doi: 10.3389/fpls.2020.00966 PMC7333729 32676094

[B59] Rahimi S. Baninasab B. Talebi M. Gholam I. M. Zarei M. (2021). Arbuscular mycorrhizal fungi inoculation improves iron deficiency in quince via alterations in host root phenolic compounds and expression of genes. Sci. Hortic-Amsterdam. 285, 110165. doi: 10.1016/j.scienta.2021.110165

[B60] Ramírez-Olvera S. M. Trejo-Téllez L. I. Gómez-Merino F. C. Ruíz-Posadas L. D. M. Alcántar-González E. G. Saucedo-Veloz C. (2021). Silicon stimulates plant growth and metabolism in rice plants under conventional and osmotic stress conditions. Plants. 10 (4), 777. doi: 10.3390/plants10040777 33920948 PMC8071275

[B61] Rashtchizadeh N. Karimi P. Dehgan P. Movahed M. S. (2015). Effects of selenium in the MAPK signaling cascade. J. Cardiovasc. Thora. Res. 7 (3), 107–112. doi: 10.15171/jcvtr.2015.23 PMC4586596 26430498

[B62] Ren L. Guo H. N. Yang J. Guo X. Y. Wei Y. S. Yang Z. (2020). Dissecting efficacy and metabolic characteristic mechanism of *Taxifolin* on renal fibrosis by multivariate approach and ultra-performance liquid chromatography coupled with mass spectrometry-based metabolomics strategy. Front. Pharmacol. 11. doi: 10.3389/fphar.2020.608511 PMC7841412 33519473

[B63] Rengel Z. Cakmak I. White P. J. (2022). Marschner’s mineral nutrition of plants (Academic Press), 2. doi: 10.1016/C2019-0-00491-8

[B64] Robinson M. D. McCarthy D. J. Smyth G. K. (2010). edgeR: a Bioconductor package for differential expression analysis of digital gene expression data. Bioinformatics. 26 (1), 139–140. doi: 10.1093/bioinformatics/btp616 19910308 PMC2796818

[B65] Rufyikiri G. Declerck S. Dufey J. E. Delvaux B. (2008). Arbuscular mycorrhizal fungi might alleviate aluminium toxicity in banana plants. New Phytol. 148 (2), 343–352. doi: 10.1046/j.1469-8137.2000.00761.x

[B66] Shahid M. Niazi N. K. Khalid S. Murtaza B. Bibi I. Rashid M. I. (2018). A critical review of selenium biogeochemical behavior in soil-plant system with an inference to human health. Environ. pollut. 234, 915–934. doi: 10.1016/j.envpol.2017.12.019 29253832

[B67] Shannon P. Markiel A. Ozier O. Baliga N. S. Wang J. T. Ramage D.. (2003). Cytoscape: A software environment for integrated models of biomolecular interaction networks. Genome. Res. 13 (11), 2498–2504. doi: 10.1101/gr.1239303 14597658 PMC403769

[B68] Shrestha J. Kandel M. Subedi S. Shah K. K. (2020). Role of nutrients in rice (*Oryza sativa* L.): A review. Agrica. 9 (1), 53–62. doi: 10.5958/2394-448X.2020.00008.5

[B69] Subramanian K. S. Balakrishnan N. Senthi N. (2013). Mycorrhizal symbiosis to increase the grain micronutrient content in maize. Aust. J. Crop Sci. 7, 900–910.

[B70] Sun C. Yang Y. Zeeshan M. Qin S. Ma J. Liu L.. (2021). Arbuscular mycorrhizal fungi reverse selenium stress in Zea mays seedlings by improving plant and soil characteristics. Ecotoxicol. Environ. Saf. 228, 113000. doi: 10.1016/j.ecoenv.2021.113000 34808506

[B71] Trippe R. C. III Pilon-Smits E. A. H. (2021). Selenium transport and metabolism in plants: Phytoremediation and biofortification implications. J. Hazard Mater. 404, 124178. doi: 10.1016/j.jhazmat.2020.124178 33068997 PMC7538129

[B72] Ueda A. Yahagi H. Fujikawa Y. Nagaoka T. Esaka M. Calcaño M.. (2013). Comparative physiological analysis of salinity tolerance in rice. Soil Sci. Plant Nutr. 59 (6), 896–903. doi: 10.1080/00380768.2013.842883

[B73] Vij S. Giri J. Dansana P. K. Kapoor S. Tyagi A. K. (2008). The receptor-like cytoplasmic kinase (OsRLCK) gene family in rice: organization, phylogenetic relationship, and expression during development and stress. Mol. Plant 1 (5), 2008. doi: 10.1093/mp/ssn047 19825577

[B74] Wang C. X. Liu X. F. Chen F. R. Yue L. Cao X. S. Li J.. (2022). Selenium content and nutritional quality of *Brassica chinensis* L enhanced by selenium engineered nanomaterials: The role of surface charge. Environ. pollut. 308, 119852. doi: 10.1016/j.envpol.2022.119582 35671896

[B75] Wu F. Y. Luo W. Q. Li J. Xing W. J. Lyu L. H. Yang J.. (2022). Effects of arbuscular mycorrhizal fungi on accumulation and translocation of selenium in winter wheat. J. Sci. Food Agric. 102 (14), 6481–6490. doi: 10.1002/jsfa.12015 35570337

[B76] Xian L. M. Li Q. R. Li T. Yu L. (2022). Methylselenized glucose: an efficient organoselenium fertilizer enhancing the selenium content in wheat grains. Chin. Chem. Lett. 34 (5), 107878. doi: 10.1016/j.cclet.2022.107878

[B77] Xie C. Mao X. Huang J. Ding Y. Wu J. Dong S.. (2011). Kobas 2.0: a web server for annotation and identification of enriched pathways and diseases. Nucleic Acids Res. 39, 316–322. doi: 10.1093/nar/gkr483 PMC3125809 21715386

[B78] Xie K. Ren Y. H. Chen A. Q. Yang C. F. Zheng Q. S. Chen J.. (2022). Plant nitrogen nutrition: The roles of arbuscular mycorrhizal fung. J. Plant Physiol. 269, 153591. doi: 10.1016/j.jplph.2021.153591 34936969

[B79] Yang D. D. Hu C. X. Wang X. Shi G. Y. Li Y. F. Fei Y. C.. (2021b). Microbes: a potential tool for selenium biofortification. Metallomics. 13 (10), mfab054. doi: 10.1093/mtomcs/mfab054 34477877

[B80] Yang C. M. Yao H. Wu Y. J. Sun G. Y. Yang W. Li Z. G.. (2021a). Status and risks of selenium deficiency in a traditional selenium-deficient area in Northeast China. Sci. Total Environ. 762, 144103. doi: 10.1016/j.scitotenv.2020.144103 33360462

[B81] Ye J. Fang L. Zheng H. Zhang Y. Chen J. Zhang Z.. (2006). WEGO: a web tool for plotting GO annotations. Nucleic Acids Res. 34 (suppl_2), W293–W297. doi: 10.1093/nar/gkl031 16845012 PMC1538768

[B82] Ye Y. M. Qu J. W. Pu Y. Xu F. Wu C. (2020). Selenium biofortification of crop food by beneficial microorganisms. J. Fungi. 6 (2), 59. doi: 10.3390/jof6020059 PMC7344654 32375266

[B83] Yuan Z. Q. Long W. X. Liang T. Zhu M. H. Zhu A. Y. Luo X. Y.. (2022). Effect of foliar spraying of organic and inorganic selenium fertilizers during different growth stages on selenium accumulation and speciation in rice. Plant Soil. 15, 1–15. doi: 10.1007/s11104-022-05567-2

[B84] Yuan J. Shi K. Zhou X. Wang L. Xu C. Zhang H.. (2023). Interactive impact of potassium and arbuscular mycorrhizal fungi on the root morphology and nutrient uptake of sweet potato (*Ipomoea batatas* L.). Front. Microbiol. 13. doi: 10.3389/fmicb.2022.1075957 PMC9869065 36699580

[B85] Zafeiriou I. Gasparatos D. Ioannou D. Massas I. (2022). Selenium uptake by lettuce plants and Se distribution in soil chemical phases affected by the application rate and the presence of a seaweed extract-based biostimulant. Soil Syst. 6 (2), 56. doi: 10.3390/soilsystems6020056

[B86] Zhang B. Horvath S. (2005). A general framework for weighted gene co-expression network analysis. Stat. Appl. Genet. Mol. Biol. 4 (1), Article17. doi: 10.2202/1544-6115.1128 16646834

[B87] Zhang M. Hu C. X. Zhao X. H. Tan Q. L. Sun X. C. Li N. (2012). Impact of molybdenum on Chinese cabbage response to selenium in solution culture. Soil Sci. Plant Nutr. 58 (5), 595–603. doi: 10.1080/00380768.2012.723603

[B88] Zhang Z. Y. Li B. Liu Y. X. He L. X. Pang T. Chen Z. D.. (2022). Arbuscular mycorrhizal fungal inoculation increases organic selenium accumulation in soybean (*Glycine max* (Linn.) Merr.) growing in selenite-spiked soils. Toxics 10 (10), 565. doi: 10.3390/toxics10100565Table 36287845 PMC9610514

[B89] Zhang J. Zhang M. Tian S. Lu L. Shohag M. J. I. Yang X. (2014). Metallothionein 2 (*SaMT2*) from *Sedum alfredii* Hance confers increased Cd tolerance and accumulation in yeast and tobacco. PloS One 9 (7), e102750. doi: 10.1371/journal.pone.0102750 25032704 PMC4102533

