# Supplementary material for: Arbuscular mycorrhizal fungi improve selenium uptake by modulating root transcriptome of rice (Oryza sativa L.)
Source: Front Plant Sci. 2023 Sep 20;14:1242463. doi: 10.3389/fpls.2023.1242463 (PMC10547891; doi:10.3389/fpls.2023.1242463)

Supplementary Material

Arbuscular mycorrhizal fungi improve selenium uptake by modulating the root transcriptome of a rice (Oryza stavia L.)

Yan Qin ^1†^, Qiuliang Cai ^2†^, Yiting Ling ^1†^, Xue Chen ^3^, Jingmao Xu ^4^, Guirong Huang ^1^, Shanhe Liang ^1^, Xiu Yuan ^1^, Xiaomu Yang ^1^,Dan Lu ^1^, Xueli Wang ^1^, Yanyan Wei ,^1^*

*** Correspondence:** Dr. Yanyan Wei: yanyanwei@gxu.edu.cn; yanyanwei2008@163.com

# Supplementary Tables

**SITable 1 qRt-PCR** **amplification procedure**

| Reaction Component | Volume |
| --- | --- |
| 2xGreen qPCR MasterMix | 10 μL |
| Forward Primer | 0.5 μL |
| Reverse Primer | 0.5 μL |
| Template DNA | 2 μL |
| RNase- Free Water | 20 μL |

**SITable 2 The parameter of OPLS-DA models. Ck: No inoculation of Fm and additional addition of Se. Fm: Inoculated only with Fm. Se: Only additional Se. Fm+Se: Both inoculated with Fm and additionally added Se**

| Treatment group | Root | | Shoot | |
| --- | --- | --- | --- | --- |
|  | R^2^Y | Q^2^ | R^2^Y | Q^2^ |
| Ck vs Fm | 0.98 | 0.92 | 0.99 | 0.96 |
| CK vs Se | 0.90 | 0.85 | 0.91 | 0.81 |
| Fm vsFm+Se | 0.95 | 0.90 | 0.95 | 0.92 |
| Se vs Fm+Se | 0.95 | 0.90 | 0.95 | 0.90 |

**SITable 3 Results of rice transcriptome gene function annotation**

|  | Gene number | Percentage（%） |
| --- | --- | --- |
| GO | 23899 | 26.64 |
| KEGG | 13105 | 14.61 |
| COG | 40384 | 45.01 |
| NR | 48339 | 53.88 |
| Swiss-Prot | 26178 | 29.18 |
| Pfam | 28972 | 32.29 |
| Total_anno | 48768 | 54.35 |
| Total | 89723 | 100 |

**SITable 4** **qRT-PCR validation primers**

| Gene ID | Primer sequences (5’-3’) |
| --- | --- |
| BGIOSGA031588 | F: GATGACCATCATCGGTGAAATG  R: CCTTCTTCTCTTCTTTTGCAGG |
| BGIOSGA036792 | F: ACGCAGAGAAACTCATGAAGTA  R: TCAAGGAATTCACTCTCAACGA |
| BGIOSGA024510 | F: CTCCTCCTTCCTTCTCAAGAAC  R: CAAGAAACCCTGCATGATGTAC |
| BGIOSGA000301 | F: CCTAAGTGGTGTGATCCTCTTC  R: GAGCATTCAGGTCAATGGAAAG |
| UBC1 | F: CCGTTTGTAGAGCCATAATTGCA  R: AGGTTGCCTGAGTCACAGTTAAGTG |

**SITable 5** **The Modules significantly associated with ions and the hub genes of highest degree in modules**

| Module | Ion | Hug gene of highest degree | Hubgene homologues |
| --- | --- | --- | --- |
| Grey60 | Zn | **BGIOSGA013928** | **-** |
| Lightyellow | Se, Mg, Al | **BGIOSGA033581** (GO:0006468 protein phosphorylation, GO:0007166 cell surface receptor signaling pathway, GO:0004674 protein serine/threonine kinase activity, GO:0004672 protein kinase activity, GO:0005524 ATP binding) | Os11g0667700 (OsRLCK348) |
| Pink | Zn | **BGIOSGA018646** | **-** |
| Green | Ni, Si, Mg, Fe | **BGIOSGA025543** | Os07g0294600 |
| Magenta | Se | **BGIOSGA021058** | Os06g0552400 |
| Royalblue | Si | **BGIOSGA027710** | Os08g0130000 (OsSTA203) |
| Salmon | Ni, Mo | **BGIOSGA034368** (GO:0046872 metal ion binding, GO:0016491 oxidoreductase activity) | Os11g0186900 (ACO4) |
| Purple | Mg, Ca, Cu, Co | **BGIOSGA005109** (GO:0016491 oxidoreductase activity) | Os01g0931000 |
| Orange | Cu | **BGIOSGA008466** (GO:0016020 membrane,GO:0016021 integral component of membrane,GO:0004497 monooxygenase activity,GO:0005506 iron ion binding,GO:0016705 oxidoreductase activity, acting on paired donors, with incorporation or reduction of molecular oxygen, GO:0016705 oxidoreductase activity, acting on paired donors, with incorporation or reduction of molecular oxygen, GO:0020037 heme binding,GO:0036202 ent-cassa-12,15-diene 11-hydroxylase activity,GO:0046872 metal ion binding) | Os02g0569900 (OsCYP76M7) |
| Lightcyan | Ca, Cu | **BGIOSGA018883** (GO:0006355 regulation of transcription, GO:0006357 regulation of transcription by RNA polymerase II, DNA-templated,GO:0005634 nucleus,GO:0003677 DNA binding,GO:0043565 sequence-specific DNA binding, GO:0000981 DNA-binding transcription factor activity, RNA polymerase II-specific) | Os05g0129700 (OsKn2) |
| Yellow | Ca, Cu | **BGIOSGA010300** (GO:0005730 nucleolus, GO:0042254 ribosome biogenesis, GO:0032040 small-subunit processome, GO:0005634 nucleus) | Os03g0598200 |
| Black | Se, Al | **BGIOSGA017551** (GO:0005622 intracellular anatomical structure, GO:0000166 nucleotide binding, GO:0004672 protein kinase activity, GO:0004674 protein serine/threonine kinase activity, GO:0004707 MAP kinase activity, GO:0005524 ATP binding, GO:0016301 kinase activity, GO:0016740 transferase activity, GO:0106310 protein serine kinase activity, GO:0000165 MAPK cascade, GO:0006468 protein phosphorylation, GO:0016310 phosphorylation | Os05g0566400 (OsMPK7) |

**SITable 6 Hug gene of black module**

| Gene id | Degree | GO | Homologue of Oryza sativa Japonica Group |
| --- | --- | --- | --- |
| BGIOSGA017551 | 108.8451 | GO:0005524 ATP binding, GO:0016740 transferase activity, GO:0004674 protein serine/threonine kinase activity, GO:0000165 MAPK cascade, GO:0006468 protein phosphorylation, GO:0016310 phosphorylation | **Os05g0566400 (OsMPK7)** |
| BGIOSGA010865 | 105.8046 | GO:0046872 metal ion binding, GO:0016020 membrane, GO:0016021 integral component of membrane | **Os03g0313300** |
| BGIOSGA004527 | 104.9461 | GO:0004714 transmembrane receptor protein tyrosine kinase activity, GO:0016740 transferase activity, GO:0004674 protein serine/threonine kinase activity, GO:0004672 protein kinase activity, GO:0000166 nucleotide binding, GO:0016301 kinase activity, GO:0005524 ATP binding, GO:0006468 protein phosphorylation, GO:0016310 phosphorylation, GO:0018108 peptidyl-tyrosine phosphorylation, GO:0016020 membrane, GO:0016021 integral component of membrane | **Os01g0769700** (**DRUS2**) |
| BGIOSGA017741 | 102.7722 | GO:0016020 membrane, GO:0016021 integral component of membrane, GO:0000139 Golgi membrane, GO:0071555 cell wall organization | **Os05g0510800 (OsCslC7)** |
| BGIOSGA016813 | 99.24384 | GO:0046872 metal ion binding, GO:0005737 cytoplasm | **Os04g0531100 (OsERG3)** |
| BGIOSGA009916 | 98.77482 | GO:0006468 protein phosphorylation, GO:0016310 phosphorylation, GO:0016740 transferase activity, GO:0004674 protein serine/threonine kinase activity, GO:0004672 protein kinase activity, GO:0000166 nucleotide binding, GO:0016301 kinase activity, GO:0005524 ATP binding | **Os03g0717000 (OsTMK)** |
| BGIOSGA005991 | 97.71439 | GO:0046872 metal ion binding, GO:0016491 oxidoreductase activity | **Os02g0630300** **(OsGA2ox9)** |
| BGIOSGA010897 | 97.63555 | GO:0005515 protein binding, GO:0005783 endoplasmic reticulum | **Os03g0304500** |
| BGIOSGA008988 | 96.85151 | GO:0004672 protein kinase activity, GO:0005524 ATP binding | **Os02g0608500** |
| BGIOSGA031227 | 93.91994 | GO:0003677 DNA binding, GO:0005634 nucleus | **Os09g0551600** |
| BGIOSGA002311 | 93.61338 |  | **Os01g0168100** |
| BGIOSGA012379 | 91.34732 | GO:0016740 transferase activity,GO:0015018 galactosylgalactosylxylosylprotein 3-beta-glucuronosyltransferase activity, GO:0016020 membrane, GO:0000139 Golgi membrane, GO:0005794 Golgi, apparatus, GO:0071555 cell wall organization | **Os03g0287800 (OsGT43B)** |
| BGIOSGA022468 | 90.761 | GO:0003677 DNA binding, GO:0005515 protein binding, GO:0009734 auxin-activated signaling pathway, GO:0006355 regulation of DNA-templated transcription, GO:0009725 response to hormone | **Os06g0196700** **(ARF16)** |

**SITable 7 Hug gene of lightyellow module**

| Gene id | Degree | GO | Homologue of Oryza sativa Japonica Group |
| --- | --- | --- | --- |
| BGIOSGA033581 | 18.25193 | GO:0006468 protein phosphorylation, GO:0007166 cell surface receptor signaling pathway, GO:0004674 protein serine/threonine kinase activity, GO:0004672 protein kinase activity, GO:0005524 ATP binding | **Os11g0667700** (**OsRLCK348**) |
| BGIOSGA037098 | 16.73508 | GO:0006351 DNA-templated transcription, GO:0000428 DNA-directed RNA polymerase complex, GO:0008270 zinc ion binding, GO:0046872 metal ion binding,GO:0003899 DNA-directed 5'-3' RNA polymerase activity, GO:0003677 DNA binding |  |
| BGIOSGA005144 | 15.3486 | GO:0005515 protein binding, GO:0016020 membrane, GO:0016021 integral component of membrane | **Os01g0942900** |
| BGIOSGA033582 | 15.20539 | GO:0005634 nucleus, GO:0005516 calmodulin binding | **Os11g0668300** |
| BGIOSGA001626 | 13.60037 | GO:0042221 response to chemical, GO:0004364 glutathione transferase activity | **OsGSTF1 Os01g0371200** **(OsGSTF1)** |
| BGIOSGA033642 | 12.50876 | GO:0004298 threonine-type endopeptidase activity,GO:0051603 proteolysis involved in protein catabolic process,GO:0019773 proteasome core complex, alpha-subunit complex,GO:0005839 proteasome core complex,GO:0000502 proteasome complex,GO:0005737 cytoplasm | **OsPAE1 Os11g0615700 (OsPAE1)** |
| BGIOSGA029060 | 11.19564 | GO:0015031 protein transport,GO:0016020 membrane,GO:0005789 endoplasmic reticulum membrane,GO:0005783 endoplasmic reticulum | **Os08g0534350** |
| BGIOSGA034707 | 11.0565 | GO:0007166 cell surface receptor signaling pathway | **OsRLCK366 Os12g0249900** **(OsRLCK366)** |

**SITable 8 Hug gene of magenta module**

| Gene id | Degree | GO | Homologue of Oryza sativa Japonica Group |
| --- | --- | --- | --- |
| BGIOSGA021058 | 96.751 |  | **Os06g0552400** |
| BGIOSGA011740 | 89.66218 | GO:0006914 autophagy, GO:0016310 phosphorylation, GO:0006468 protein phosphorylation, GO:0005776 autophagosome, GO:0005524 ATP binding, GO:0016301 kinase activity, GO:0000166 nucleotide binding, GO:0004672 protein kinase activity, GO:0004674 protein serine/threonine kinase activity, GO:0016740 transferase activity | **Os03g0122000** |
| BGIOSGA020371 | 85.70628 | GO:0003677 DNA binding, GO:0003700 DNA-binding transcription factor activity, GO:0043565 sequence-specific DNA binding, GO:0006355 regulation of transcription, DNA-templated, GO:0050896 response to stimulus,GO:0005634 nucleus;WRKY transcription factor 49 | **Os05g0565900** **(OsWRKY49)** |
| BGIOSGA007482 | 85.10078 | GO:0005515 protein binding,GO:0043130 ubiquitin binding, GO:0009507 chloroplast | **Os02g0135900** |
| BGIOSGA007726 | 83.24731 | GO:0006952 defense response, GO:0006351 DNA-templated transcription, GO:0006355 regulation of DNA-templated transcription, GO:0003677 DNA binding, GO:0003700 DNA-binding transcription factor activity, GO:0043565 sequence-specific DNA binding | **OsbZIP17 Os02g0194900** |
| BGIOSGA025502 | 82.92179 | GO:0046872 metal ion binding | **Os07g0272400** |
| BGIOSGA023323 | 82.15786 |  | **Os06g0636100** |
| BGIOSGA037800 | 81.84546 | GO:0009941 chloroplast envelope, GO:0016021 integral component of membrane, GO:0016020 membrane, GO:0009536 plastid,GO:0015299 solute:proton antiporter activity,GO:0006812 cation transport,GO:1902600 proton transmembrane transport,GO:0055085 transmembrane transport | **Os12g0616500** **(OsCHX15)** |
| BGIOSGA000177 | 81.22463 | GO:0005515 protein binding | **Os01g0934000** **(OsATG18c)** |
| BGIOSGA022129 | 79.19494 | GO:0016747 acyltransferase activity, transferring groups other than amino-acyl groups, GO:0050734 hydroxycinnamoyltransferase activity | **Os06g0103200** |
| BGIOSGA013962 | 78.76529 | GO:0016788 hydrolase activity, acting on ester bonds | **Os03g0852800** **(OsNPC3)** |
| BGIOSGA008489 | 78.3811 | GO:0016757 glycosyltransferase activity, GO:0016758 hexosyltransferase activity, GO:0016740 transferase activity,GO:0008194 UDP-glycosyltransferase activity | **Os02g0578300** |
| BGIOSGA022893 | 77.79234 | GO:0090501 RNA phosphodiester bond hydrolysis,GO:0090502 RNA phosphodiester bond hydrolysis, endonucleolytic, GO:0006396 RNA processing,GO:0003723 RNA binding,GO:0004525 ribonuclease III activity,GO:0003723 RNA binding | **Os06g0358800** |
| BGIOSGA007749 | 77.68184 | GO:0070,417 cellular response to cold, GO:0016036 cellular response to phosphate starvation, GO:0051511 negative regulation of unidimensional cell growth, GO:2000024 regulation of leaf development, GO:0005634 nucleus | **Os02g0202200** **(OsSPX2)** |
| BGIOSGA019109 | 77.34415 | GO:0051082 unfolded protein binding, GO:0006457 protein folding, GO:0005783 endoplasmic reticulum |  |
| BGIOSGA003379 | 76.78821 | GO:0006744 ubiquinone biosynthetic process, GO:0010224 response to UV-B, GO:0017004 cytochrome complex assembly, GO:0015996 chlorophyll catabolic process | **Os01g0318700** |

# Supplementary Figures

**SIFigure 1** Principal component analysis (PCA) of ions in (A) roots and (B) shoots

**SIFigure 2** Key ions of roots in comparison groups, (A) Ck vs Fm, (B) Ck vs Se, (C) Fm vs Fm+Se, (D) Se vs Fm+Se.

**SIFigure 3** Key ions of shoots in comparison groups, (A) Ck vs Fm, (B) Ck vs Se, (C) Fm vs Fm+Se, (D) Se vs Fm+Se.

**SIFigure 4** Correlation analysis of ions in (A) roots and (B) shoots.

**SI Figure 1**


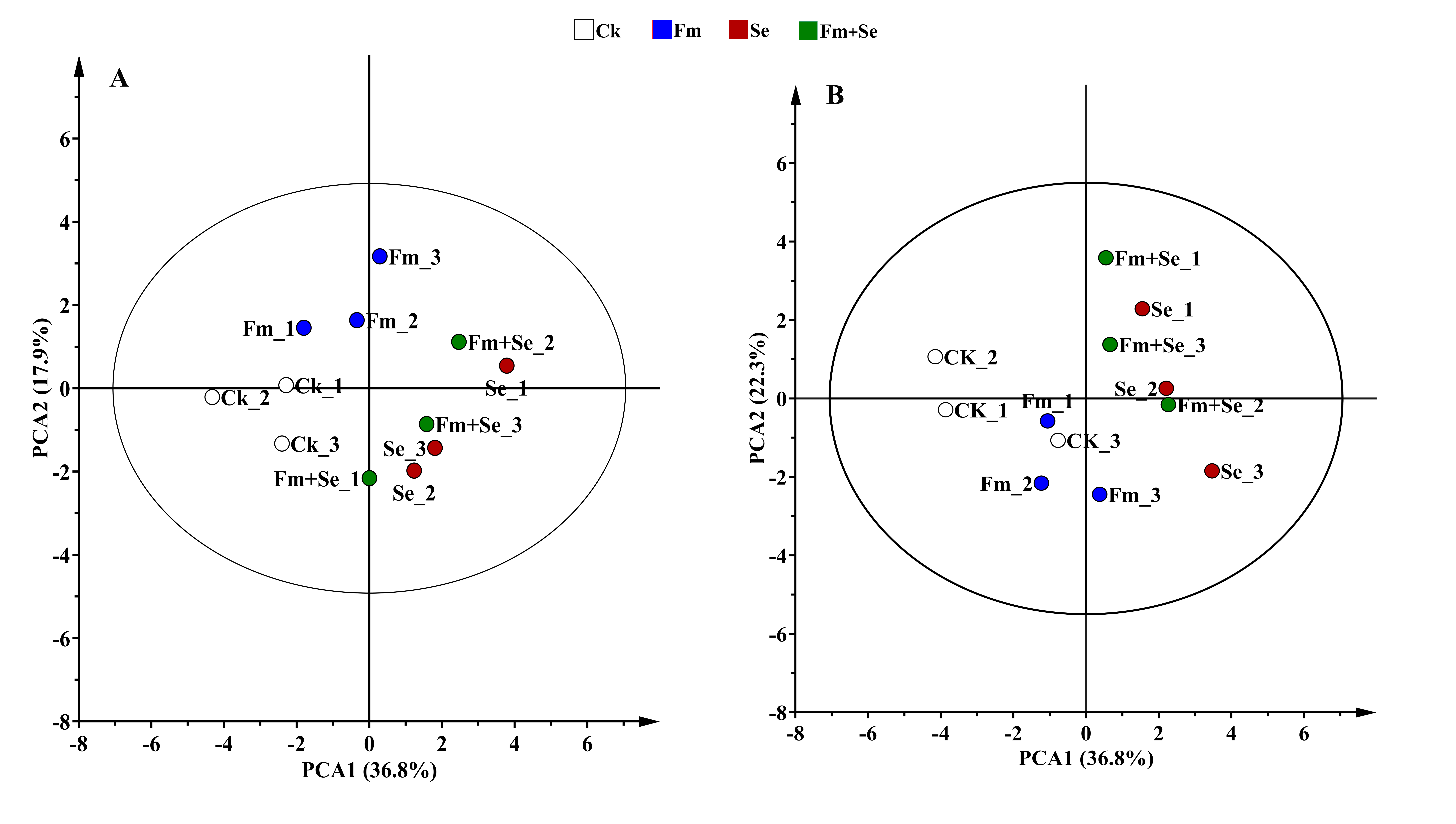


**SIFigure 2**


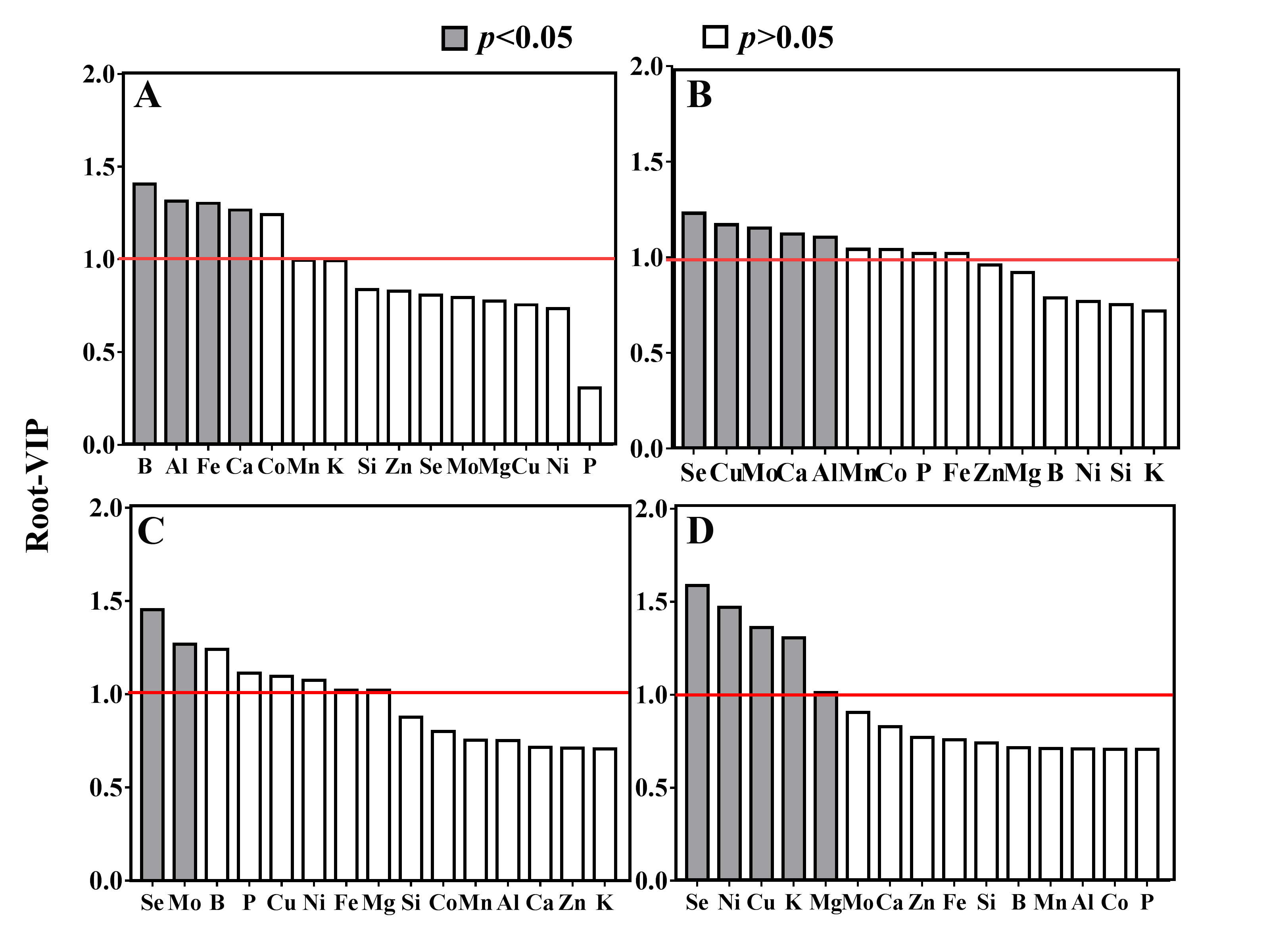


**SI Figure 3**


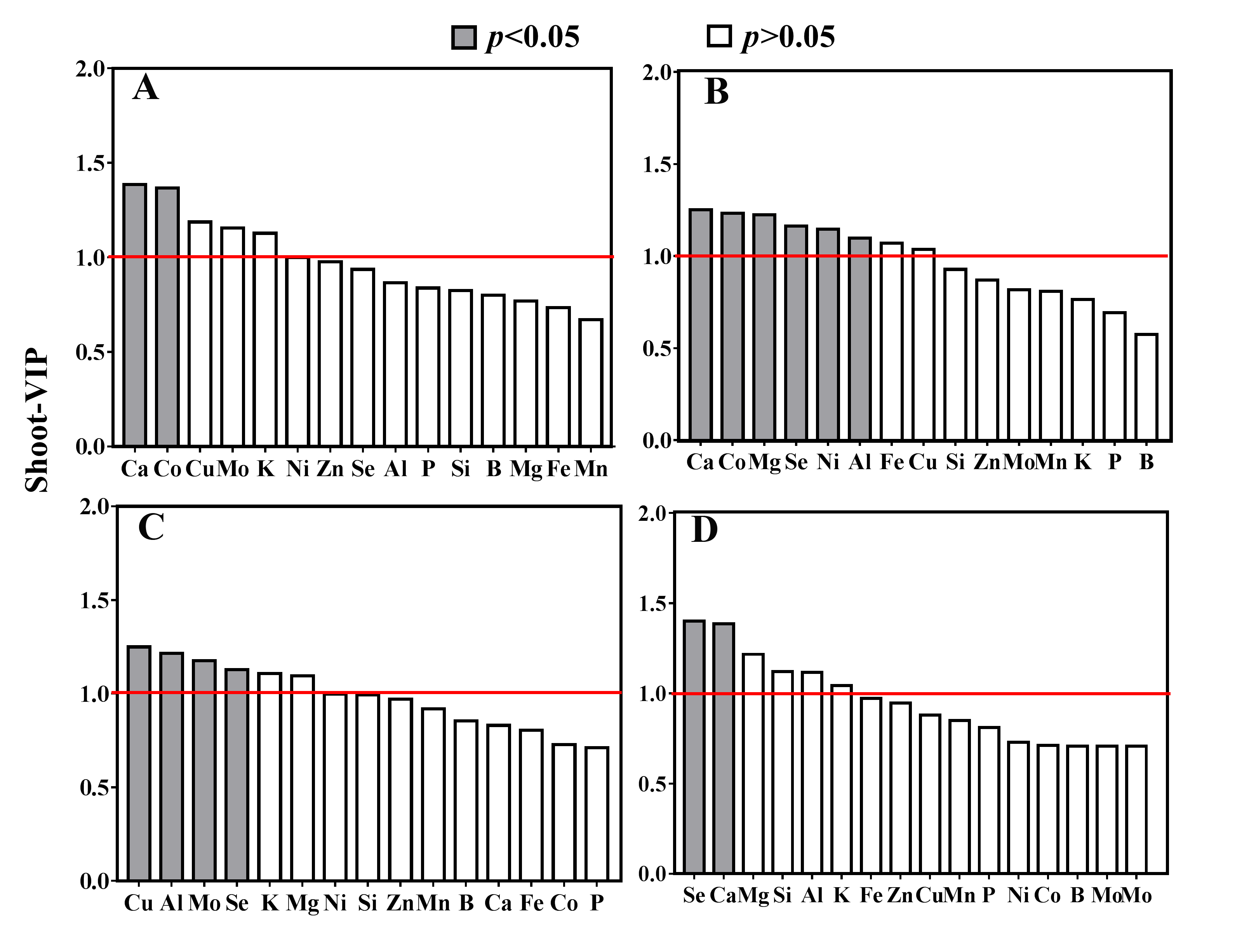


**SIFigure 4**


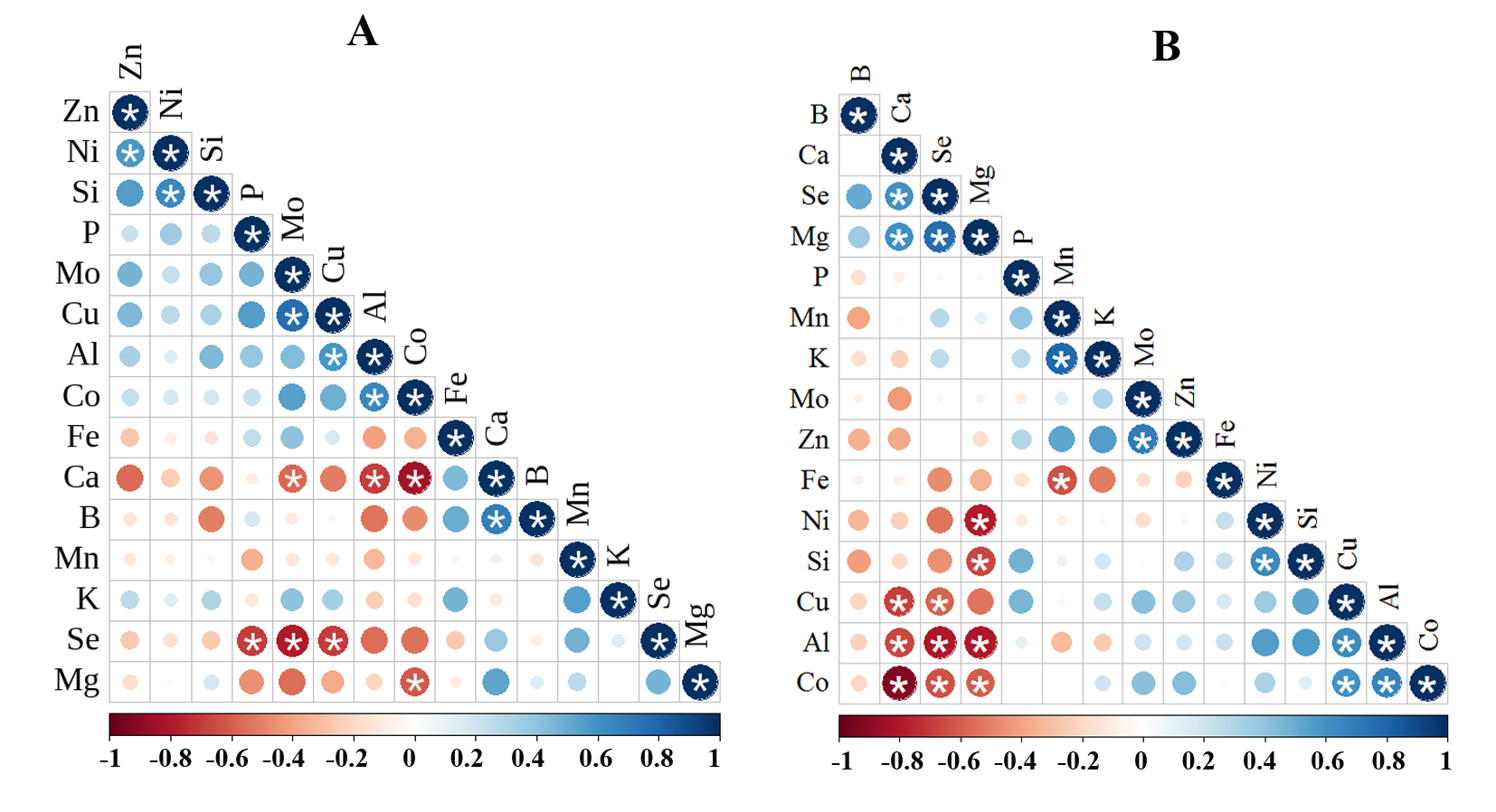

Supplement: Supplementary file 1 [file DataSheet_1.docx]
